# Diversity and Habitat Preferences of Cultivated and Uncultivated Aerobic Methanotrophic Bacteria Evaluated Based on *pmoA* as Molecular Marker

**DOI:** 10.3389/fmicb.2015.01346

**Published:** 2015-12-15

**Authors:** Claudia Knief

**Affiliations:** Institute of Crop Science and Resource Conservation – Molecular Biology of the Rhizosphere, University of BonnBonn, Germany

**Keywords:** methanotrophic bacteria, *pmoA*, diversity, phylogeny, habitat specificity, ecological niche

## Abstract

Methane-oxidizing bacteria are characterized by their capability to grow on methane as sole source of carbon and energy. Cultivation-dependent and -independent methods have revealed that this functional guild of bacteria comprises a substantial diversity of organisms. In particular the use of cultivation-independent methods targeting a subunit of the particulate methane monooxygenase (*pmoA*) as functional marker for the detection of aerobic methanotrophs has resulted in thousands of sequences representing “unknown methanotrophic bacteria.” This limits data interpretation due to restricted information about these uncultured methanotrophs. A few groups of uncultivated methanotrophs are assumed to play important roles in methane oxidation in specific habitats, while the biology behind other sequence clusters remains still largely unknown. The discovery of evolutionary related monooxygenases in non-methanotrophic bacteria and of *pmoA* paralogs in methanotrophs requires that sequence clusters of uncultivated organisms have to be interpreted with care. This review article describes the present diversity of cultivated and uncultivated aerobic methanotrophic bacteria based on *pmoA* gene sequence diversity. It summarizes current knowledge about cultivated and major clusters of uncultivated methanotrophic bacteria and evaluates habitat specificity of these bacteria at different levels of taxonomic resolution. Habitat specificity exists for diverse lineages and at different taxonomic levels. Methanotrophic genera such as *Methylocystis* and *Methylocaldum* are identified as generalists, but they harbor habitat specific methanotrophs at species level. This finding implies that future studies should consider these diverging preferences at different taxonomic levels when analyzing methanotrophic communities.

## Occurrence and role of methane-oxidizing bacteria

The activity of methane-oxidizing bacteria contributes significantly to the global methane budget. Methane is the second most abundant carbon compound in the atmosphere with a current concentration of 1.8 ppmv and a 26-fold stronger radiative efficiency compared to carbon dioxide (IPCC, 2013). The major sink of atmospheric methane is its oxidation by OH radicals, but soils also serve as sink by about 5% due to the activity of methanotrophic bacteria (IPCC, 2013). Moreover, methanotrophs are of particular importance in attenuating net fluxes of this greenhouse gas into the atmosphere in diverse ecosystems that are sources of atmospheric methane (De Visscher et al., 2007; Reeburgh, 2007; Conrad, 2009). Known sources are freshwater and permafrost ecosystems, some animal species and termites, and the release of methane from geological processes, wildfires and hydrates. Another 50–65% of the total emissions are due to anthropogenic activities including ruminant husbandry, fossil fuel extraction and use, rice paddy agriculture and emissions from landfills and waste, resulting in a current elevation of the atmospheric methane concentration by a factor of 2.5 compared to preindustrial times (IPCC, 2013). All these ecosystems with source function for atmospheric methane are typical habitats of methane-oxidizing bacteria. These include freshwater and marine sediments and water columns, aquifers, floodplains, peat bogs, high-arctic, and tundra wetlands, upland soils, rice paddies, landfill covers, and sewage sludge (Hanson and Hanson, 1996; Conrad, 2007; Bowman, 2014).

Besides their importance in the global methane cycle, aerobic methanotrophic bacteria are of biotechnological interest since a long time. They can be used for biodegradation processes of organic pollutants based on the fact that the key enzyme for methanotrophy in these organisms, the methane monooxygenase, catalyzes diverse non-specific oxidation reactions, e.g., of chlorinated solvents such as trichloroethylene (Hanson and Hanson, 1996; Smith and Dalton, 2004; Dalton, 2005; Jiang et al., 2010; Semrau et al., 2010; Strong et al., 2015). Moreover, methanotrophs have been studied in detail with regard to their potential to convert methane to complex organic molecules of higher value. Since the 1970s, methanotrophic bacteria have been studied for single cell protein production (Dalton, 2005). Besides, biopolymers such as polyhydroxybutyrate, metabolic products such as organic acids, vitamins, pigments or lipids (for biodiesel production) may be produced from methane by methanotrophs (Strong et al., 2015). Further possible applications for biosynthesis processes are based on the co-metabolic activities of the methane monooxygenase, e.g., for epoxide production via the conversion of propene to epoxypropane (Hanson and Hanson, 1996; Dalton, 2005). Moreover, researchers address the question to what extent methanotrophic bacteria can be used to increase reduction of methane emissions from anthropogenic sources such as landfills or coal mines (Jiang et al., 2010).

## Diversity and ecophysiology of cultivated methanotrophic bacteria

### Brief history about the cultivation of aerobic methanotrophic bacteria and current diversity and phylogeny of cultivated methanotrophs

Methanotrophic bacteria have been studied since the beginning of the last century, initiated by the work of Kaserer (1905) and Söhngen (1906) who reported for the first time the existence of methane-oxidizing bacteria. The first isolates were methanotrophic *Gammaproteobacteria*, among them *Methylomonas methanica*, initially referred to as “*Bacillus methanicus”* (Söhngen, 1906), and *Methylococcus capsulatus* (Foster and Davis, 1966). Extensive enrichment and isolation work by Whittenbury et al. (1970b) led to isolates of further *Gammaproteobacteria* and the genera *Methylocystis* and *Methylosinus*, i.e., the first methanotrophic *Alphaproteobacteria*. During the following years and with the availability of molecular methods for the rapid identification and classification of bacteria, several existing strains were reclassified and new genera were described (e.g., Bowman et al., 1993, 1995; Bodrossy et al., 1997). In particular the work of the last 10 years has resulted in a doubling of the number of known genera and species. Currently, 18 genera of cultivated aerobic methanotrophic *Gammaproteobacteria* and 5 genera of *Alphaproteobacteria* are known, represented by approx. 60 different species (Table 1). The number of *Gammaproteobacteria* increases to 20 if “*Candidatus* Crenothrix polyspora” and “*Candidatus* Clonothrix fusca” are included. These genera do not contain cultivated representatives but were only studied in natural enrichments so far (Stoecker et al., 2006; Vigliotta et al., 2007). To give an exact number of known methanotrophic taxa at species level is difficult because the taxonomic status of some species, e.g., “*Methylomonas rubra*,” *Methylococcus chroococcus, Methylococcus mobilis* or *Methylococcus thermophilus* is unclear (Table 2). In addition to the species considered in this review, more species have been described in the (early) literature, in particular within the genera *Methylomonas* and *Methylocystis* (e.g. Whittenbury et al., 1970b; Gal'chenko et al., 1977), but these were never validated. Several of them will probably be members of species that have been described in the meantime. For an overview of non-validated species with uncertain taxonomic position the reader is referred to Green (1992) or the relevant chapters in taxonomic textbooks (Bowman, 2005a,b, 2014).

**Table 1:** **Taxonomic and physiological characteristics of aerobic methanotrophic ***Alphaproteobacteria*****.

**Type strain^a^ Culture collection numbers**	**Type**	**Isolation source^b^**	**(Eco-) physiology**	**pMMO present**	***mmoX* sequence**	**References^c^**
***Methylocystaceae***						
*Methylocystis bryophila* H2s DSM 21852, VKM B-2545	IIa	Peat bog lake	Facultative methanotrophic, moderate acidophilic	Yes	FN422005	Belova et al., 2013
*Methylocystis echinoides* 2 IMET 10491, LMG 27198, NCIMB 13100, UNIQEM 25, VKM B-2128	IIa	Sewage treatment plant	Facultative methanotroph	Yes	Not detected	Gal'chenko et al., 1977; Bowman et al., 1993
*Methylocystis heyeri* H2 DSM 16984, VKM B-2426	IIa	Peat bog lake	Facultative methanotrophic, moderate acidophilic	Yes	AM283545	Dedysh et al., 2007
*Methylocystis hirsuta* CSC1 ATCC BAA-1344, DSM 18500, LMG 27832	IIa	Groundwater		Yes	DQ664498	Lindner et al., 2007
*Methylocystis parvus* OBBP ^*^ ACM 3309, ATCC 35066, IMET 10483, NCIMB 11129, UNIQEM 38, VKM B-2129	IIa	(Soil and fresh water sediments)		Yes	Not detected	Whittenbury et al., 1970b; Bowman et al., 1993; del Cerro et al., 2012
*Methylocystis rosea* SV97 ATCC BAA-1196, DSM 17261, LMG 27835	IIa	Arctic wetland soil		Yes	Not detected	Wartiainen et al., 2006b
*Methylosinus sporium* 5 ACM 3306, ATCC 35069, DSM 17706, IMET 10545, NCIMB 11126, UNIQEM 60	IIa	Rice paddy		Yes	DQ386732	Whittenbury et al., 1970b; Bowman et al., 1993
*Methylosinus trichosporium* OB3b^*^ ACM 3311, ATCC 35070, IMET 10543, NCIMB 11131, UNIQEM 75, VKM B-2117	IIa	(Soil, fresh water sediments, groundwater)		Yes	X55394	Whittenbury et al., 1970b; Bowman et al., 1993; Stein et al., 2010
***Beijerinckiaceae***						
*Methylocapsa acidiphila* B2 ^*^ DSM 13967, NCIMB 13765	IIb	Peat bog	Moderate acidophilic	Yes	Not detected	Dedysh et al., 2002; Tamas et al., 2014
*Methylocapsa aurea* KYG DSM 22158, VKM B-2544	IIb	Forest soil	Moderate acidophilic, facultative methanotrophic	Yes	Not detected	Dunfield et al., 2010
*Methylocapsa palsarum* NE2 LMG 28715, VKM B-2945	IIb	*Sphagnum* from a palsa	Moderate acidophilic	Yes	Not detected	Dedysh et al., 2015a
*Methylocella palustris* K^*^ ATCC 700799	IIb	Peat bog	Moderate acidophilic, facultative methanotrophic	No	AJ458535	Dedysh et al., 2000
*Methylocella silvestris* BL2 CIP 108128, DSM 15510, LMG 27833, NCIMB 13906	IIb	Forest soil	Moderate acidophilic, facultative methanotrophic	No	AJ491848	Dunfield et al., 2003; Chen et al., 2010
*Methylocella tundrae* T4 DSM 15673, LMG 27838, NCIMB 13949	IIb	Tundra peatland	Moderate acidophilic	No	AJ555245	Dedysh et al., 2004
*Methyloferula stellata* AR4 ^*^ DSM 22108, LMG 25277, VKM B-2543	IIb	Peat bog	Moderate acidophilic	No	FR686346	Vorobev et al., 2011; Dedysh et al., 2015b

a *Type species representing the respective genus are marked with an asterisk*.

b *Information given in brackets refers to information obtained from the analysis of a related strain, since this information was not available for the type strain of the species*.

c *Listed publications describe the initial isolation, current classification and genome sequence of the strain as far as already available*.

**Table 2:** **Taxonomy, isolation source, and physiological characteristics of aerobic methanotrophic ***Gammaproteobacteria*****.

**Type strain^a^ Culture collection numbers**	**Type**	**Isolation source^b^**	**(Eco-) physiology**	***mmoX* sequence**	**Synonym/basonym**	**References^c^**
***Methylococcaceae***						
*Methylobacter luteus* 53v^*^ ACM 3304, ATCC 49878, IMET 10584, NCIMB 11914, UCM 53B, VKM 53B	Ia	Sewage		Not analyzed	*Methylococcus luteus* *Methylobacter bovis* *Methylococcus bovis* *Methylococcus fulvus*	Romanovskaya et al., 1978; Bowman et al., 1993
*Methylobacter marinus* A45 ACM 4717	Ia	Seawater sediment		Not detected	*Methylomonas marinus*	Lidstrom, 1988; Bowman et al., 1993
*Methylobacter psychrophilus* Z-0021 VKM B-2103	Ia	Tundra	Psychrophilic	Not analyzed		Omelchenko et al., 1996
*Methylobacter tundripaludum* SV96 ATCC BAA-1195, DSM 17260	Ia	Tundra soil		Not detected		Wartiainen et al., 2006a; Svenning et al., 2011
*Methylobacter whittenburyi* Y ACM 3310, ATCC 51738, NCIMB 11128	Ia	Lake sediment		Not analyzed	*Methylobacter capsulatus* *Methylococcus whittenburyi* *Methylobacter vinelandii* *Methylococcus vinelandii*	Romanovskaya et al., 1978; Bowman et al., 1993
*Methyloglobulus morosus* KoM1 ^*^ DSM 22980, JCM 18850	Ia	Lake sediment	Perferentially microaerophilic	Not detected		Poehlein et al., 2013; Deutzmann et al., 2014
*Methylomarinum vadi* IT-4^*^ DSM 18976, JCM 13665	Ia	Marine hydrothermal system	Halophilic	Not detected		Hirayama et al., 2013
*Methylomicrobium agile* A30 ^*^ ACM 3308, ATCC 35068, NCIMB 11124	Ia	Sewage		Not detected	*Methylomonas agile* *Methylobacter agilis*	Whittenbury et al., 1970b; Bowman et al., 1995; Hamilton et al., 2015
*Methylomicrobium album* BG8 ACM 3314, ATCC 33003, NCIMB 11123, VKM-BG8	Ia	Soil		Not detected	*Methylomonas albus Methylobacter albus*	Whittenbury et al., 1970b; Bowman et al., 1995; Kits et al., 2013
*Methylomicrobium alcaliphilum* 20Z DSM 19304, LMG 27836, NCIMB 14124, VKM B-2133	Ia	Soda lake	Moderate halophilic, alkaliphilic	Not detected	*Methylobacter alcaliphilus*	Khmelenina et al., 1997; Kalyuzhnaya et al., 2008; Vuilleumier et al., 2012
*Methylomicrobium buryatense* 5B VKM B-2245	Ia	Soda lake	Moderate halophilic	AOTL00000000		Kaluzhnaya et al., 2001; Khmelenina et al., 2013
*Methylomicrobium japanense* NI FERM BP-5633, NBRC 103677, VKM B-2462	Ia	Marine sediment	Slightly halophilic	AB253366		Kalyuzhnaya et al., 2008
*Methylomicrobium kenyense* AMO1 DSM 19305, NCCB 97157, NCIMB 13566, VKM B-2464	Ia	Soda lake	Moderate halophilic, alkaliphilic	Not detected		Kalyuzhnaya et al., 2008
*Methylomicrobium pelagicum* AA-23 ACM 3505, NCIMB 2265	Ia	Marine	Moderate halophilic	Not detected	*Methylomonas pelagica* *Methylobacter pelagicus*	Sieburth et al., 1987; Bowman et al., 1995
*Methylomonas aurantiaca* JB103 ACM3406, UQM 3406	Ia	Sewage		Not analyzed		Bowman et al., 1990
“*Methylomonas denitrificans”* FJG	Ia	Soil		Not detected		Kits et al., 2015
*Methylomonas fodinarum* LD2 ACM3268, UQM 3268	Ia	Coal mine drainage water		Not analyzed		Bowman et al., 1990
*Methylomonas koyamae* Fw12E-Y JCM 16701, LMG 26899, NBRC 105905, NCIMB 14606	Ia	Rice paddy		Not detected		Ogiso et al., 2012
*Methylomonas lenta* R-45377 LMG 26260, JCM 19378	Ia	Manure		Not detected		Hoefman et al., 2014a
*Methylomonas methanica* S_1_^*^ ACM 3307, ATCC 35067, IMET10543, NCIMB 11130, UNIQEM 8, VKM B-2110	Ia	(Freshwater sediment, lake, pond water, swamply soil)		Not detected	*Bacillus methanicus* *Methanomonas methanica* *Pseudomonas methanica*	Söhngen, 1906; Whittenbury and Krieg, 1984
*Methylomonas paludis* MG30 DSM 24973, VKM B-2745	Ia	Peat bog	Acid-tolerant	Not detected		Danilova et al., 2013
“*Methylomonas rubra”* 15m^d^ ACM3303, NCIMB11913, UCM B-3075, VKM-15m	Ia	Coal mine drainage water		Not analyzed	*Methylomonas rubrum*	Whittenbury et al., 1970b; Romanovskaya et al., 2006
*Methylomonas scandinavica* SR5 VKM B-2140	Ia	Groundwater	Psychrotolerant	Not detected		Kalyuzhnaya et al., 1999
*Methyloprofundus sedimenti* WF1 ^*^ ATCC BAA-2619, LMG 28393	Ia	Marine sediment	Moderate halophilic	Not detected		Tavormina et al., 2015
*Methylosarcina fibrata* AML-C10 ^*^ ATCC 700909, DSM 13736	Ia	Landfill cover soil		Not detected		Wise et al., 2001; Hamilton et al., 2015
*Methylosarcina lacus* LW14 ATCC BAA-1047, DSM 16693, JCM 13284	Ia	Lake		Not detected		Kalyuzhnaya et al., 2005, 2015
*Methylosarcina quisquiliarum* AML-D4 ATCC 700908, DSM 13737	Ia	Landfill cover soil		Not detected		Wise et al., 2001
*Methylosoma difficile* LC2 ^*^ DSM 18750, JCM 14076	Ia	Lake sediment	Microaerobic	Not detected		Rahalkar et al., 2007
*Methylosphaera hansonii* AM6 ^*^ ACAM 549	Ia	Lake	Psychrophilic, moderate halophilic	Not detected		Bowman et al., 1997
*Methylovulum miyakonense* HT12^*^ ATCC BAA-2070, DSM 23269, NBRC 106162	Ia	Forest soil		AB501288		Iguchi et al., 2011; Hamilton et al., 2015
*Methylocaldum gracile* 14L NCIMB 11912, VKM-14L	Ib	Fresh water mud	Thermotolerant	Not detected	*Methylomonas gracilis*	Romanovskaya et al., 1978; Bodrossy et al., 1997
*Methylocaldum marinum* S8 DSM 27392, NBRC 109686	Ib	Marine sediment	Thermotolerant, moderate halophilic	AB900160		Takeuchi et al., 2014
*Methylocaldum szegediense* OR2^*^	Ib	Water from hot spring	Moderate thermophilic	Not detected		Bodrossy et al., 1997
*Methylocaldum tepidum* LK6	Ib	Agricultural soil	Thermotolerant	Not detected		Bodrossy et al., 1997
*Methylococcus capsulatus* Texas^*^ ACM1292, ATCC 19069, NCIMB 11853, UNIQEM 1	Ib	Sewage sludge	Thermotolerant	AMCE00000000		Foster and Davis, 1966; Kleiveland et al., 2012
*Methylococcus chroococcus 9*^e^	Ib	(Soil, fresh water sediments, groundwater)		Not analyzed	*Methylobacter chroococcum*?	Whittenbury et al., 1970b; Romanovskaya et al., 1978
*Methylococcus mobilis* LMD 77.28^e^ NCCB 77028	Ib	Active silt		Not analyzed		Hazeu et al., 1980
*Methylococcus thermophilus* VKM-2Yu^f^ ACM3585, IMV-B 3037	Ib	(Mud, soils, pond sediment)	Thermophilic	Not analyzed		Malashenko et al., 1975
*Methylogaea oryzae* E10 ^*^ DSM 23452, JCM 16910	Ib	Rice paddy		Not detected		Geymonat et al., 2010
*Methylomagnum ishizawai* RS11D-Pr DSM 29768, JCM 18894, KCTC 4681, NBRC 109438	Ib	Rice rhizosphere		AB983338		Khalifa et al., 2015
*Methyloparacoccus murrellii* R-49797 ^*^ LMG 27482, JCM 19379	Ib	Pond water		Not detected		Hoefman et al., 2014b
“*Candidatus* Clonothrix fusca”^g^ (no pure culture)		Drinking water		Not analyzed		Vigliotta et al., 2007
***Methylothermaceae***						
*Methylohalobius crimeensis* 10Ki^*^ ATCC BAA-967, DSM 16011	Ic	Hypersaline lake sediment	Moderate halophilic	Not detected		Heyer et al., 2005; Sharp et al., 2015
*Methylomarinovum caldicuralii* IT-9 ^*^ DSM 19749, JCM 13666	Ic	Marine hydrothermal system	Moderate thermophilic, moderate halophilic	Not detected		Hirayama et al., 2014
*Methylothermus subterraneus* HTM55 DSM 19750, JCM 13664	Ic	Hot aquifer	Moderate thermophilic	Not detected		Hirayama et al., 2010
*Methylothermus thermalis* MYHT^*^ IPOD FERM P-19714, VKM B-2345	Ic	Hot spring	Moderate thermophilic, halotolerant	Not detected		Tsubota et al., 2005
***Crenotrichaceae***						
“*Candidatus* Crenothrix polyspora”^g^ (no pure culture)		Drinking water	Facultative methanotrophic	Not detected		Stoecker et al., 2006

a *Type species representing the respective genus are marked with an asterisk*.

b *Information given in brackets refers to information obtained from the analysis of a related strain, since this information was not available for the type strain of the species*.

c *Listed publications describe the initial isolation, current classification and genome sequence of the strain as far as already available*.

d *The type strain of Methylomonas rubra was reported to belong to the species “Methylomonas methanica” (Bowman et al., 1993), but later it was proposed to represent a separate species (Romanovskaya et al., 2006); this has not been validated yet*.

e *According to Bergey's manual of systematic bacteriology, strains of these species do not exist anymore and the species are not considered as valid (Bowman, 2005a), but at least for Methylococcus mobilis, this appears not to be correct, as the type strain is listed in the NCCB catalog*.

f *According to Bodrossy et al. (1997), the type strain is no longer available and the available strain M. thermophilus strain IMV-B3122 (NCIMB 13419) is actually a strain of Methylocaldum tepidum*.

g *Candidatus Crenothrix polyspora and Clonothrix fusca do not contain any cultured type strains and the genus Clonothrix is not validated according to the list of prokaryotic names with standing in nomenclature (Parte, 2014), therefore these genera are referred to as Candidatus; according to Op den Camp et al. (2009) the validated family Crenotrichaceae is phylogenetically a subset of the Methylococcaceae*.

The known diversity of aerobic methanotrophic bacteria was further expanded by the detection of methanotrophic bacteria within the phylum *Verrucomicrobia* (Table 3). Their existence was described in three independent studies in 2007 and 2008 (Dunfield et al., 2007; Pol et al., 2007; Islam et al., 2008) and they were reported to represent distinct species of the genus “*Methylacidiphilum”* (Op den Camp et al., 2009). Recently, a second genus within the newly formed methanotrophic family *Methylacidiphilaceae* was proposed, “*Methylacidimicrobium*,” also consisting of three species (van Teeseling et al., 2014).

**Table 3:** **Isolation source and physiological characteristics of methanotrophic bacteria harboring ***pmoA*** outside the phylum ***Proteobacteria*****.

**Type strain**	**Type**	**Isolation source**	**(Eco-)physiology**	***mmoX* sequence**	**Synonym/basonym**	**References^a^**
***Verrucomicrobia, Methylacidiphilaceae***						
“*Methylacidimicrobium cyclopophantes”* 3B	III	Volcanic soil	Thermophilic, acidophilic	Not detected		van Teeseling et al., 2014
“*Methylacidimicrobium fagopyrum”* 3C	III	Volcanic soil	Thermophilic, acidophilic	Not detected		van Teeseling et al., 2014
“*Methylacidimicrobium tartarophylax”* 4AC	III	Volcanic soil	Thermophilic, acidophilic	Not detected		van Teeseling et al., 2014
“*Methylacidiphilum fumariolicum”* SolV	III	Themal mud pod	Thermophilic, acidophilic	Not detected	“*Acidimethylosilex fumarolicum”*	Pol et al., 2007; Op den Camp et al., 2009; Khadem et al., 2012
“*Methylacidiphilum infernorum”* V4	III	Soil, geothermal area	Thermophilic, acidophilic	Not detected	“*Methylokorus infernorum”*	Dunfield et al., 2007; Hou et al., 2008; Op den Camp et al., 2009
“*Methylacidiphilum kamchatkense”* Kam1	III	Hot spring	Thermophilic, acidophilic	Not detected	“*Methyloacida kamchatkensis”*	Islam et al., 2008; Op den Camp et al., 2009; Erikstad and Birkeland, 2015
**CANDIDATE DIVISION NC10**						
“*Candidatus* Methylomirabilis oxyfera” NC10	–	River sediment	Anaerobic	Not detected		Ettwig et al., 2010

a *Listed publications describe the initial isolation, current classification and genome sequence of the strain as far as available*.

**Figure 1:**
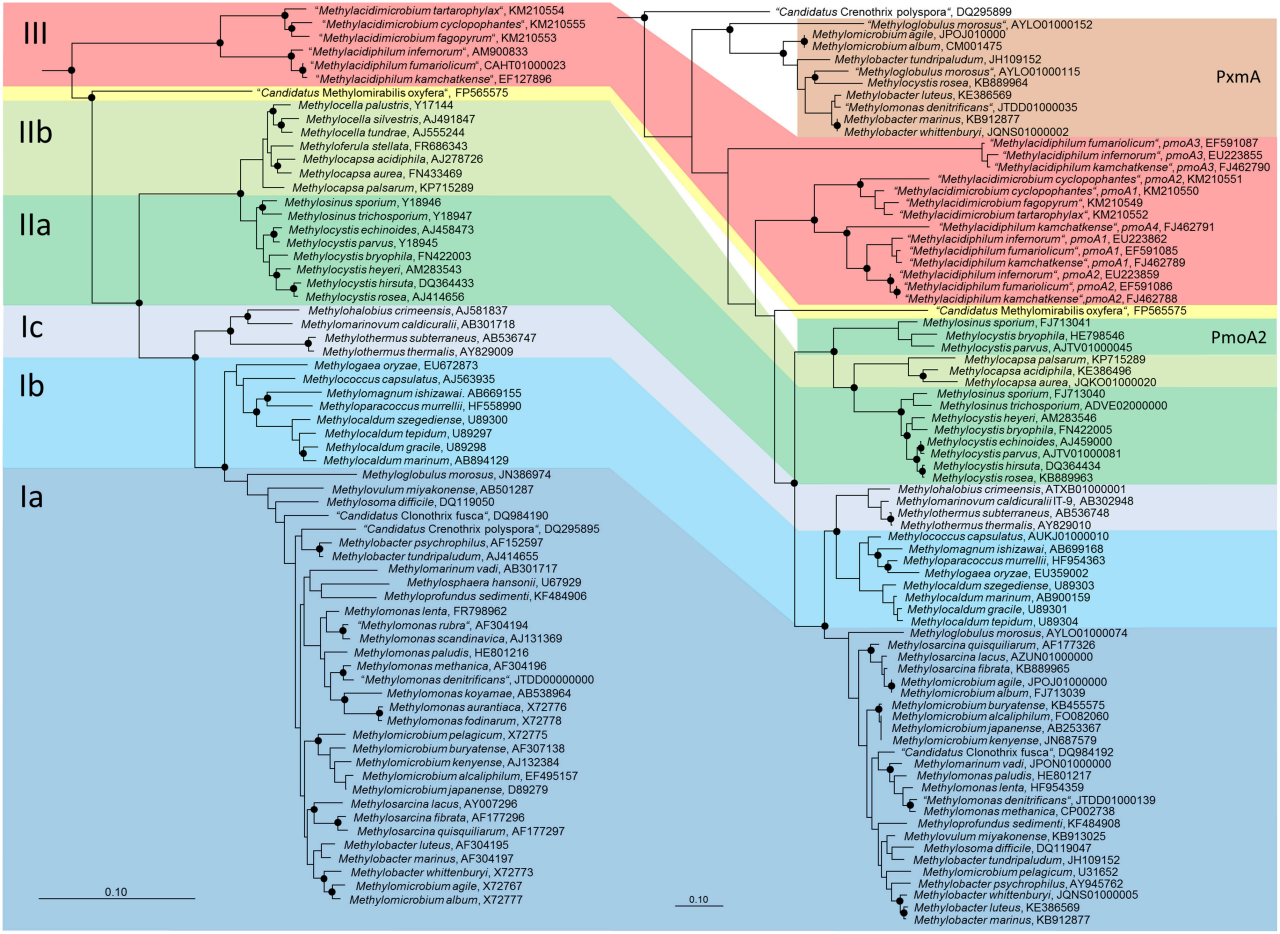
**Phylogenetic trees showing the phylogeny of methanotrophic type strains based on 16S rRNA gene sequences (left tree) and PmoA sequences (right tree)**. The neighbor joining trees were calculated using the ARB software package (Ludwig et al., 2004) based on 1556 nucleotide positions with Jukes Cantor correction or 160 amino acid positions with Kimura correction, respectively. PmoA sequences of *Methylobacter luteus, Methylobacter whittenburyi*, and *Methylomicrobium pelagicum* are not available from the type strains, but were taken from a different strain representing the species. The 16S rRNA gene based tree was rooted with sequences of methanogenic *Archaea* (AB301476, M60880, AB065296, AM114193, AB196288), the PmoA tree with AmoA sequences of ammonia-oxidizing bacteria (NC_004757, X90822). Dots label branch points that were confirmed in maximum likelihood trees. The scale bars display 0.10 changes per nucleotide or amino acid position.

Phylogenetically, the methanotrophic *Alphaproteobacteria* belong to two families, the *Methylocystaceae* and *Beijerinckiaceae* (Figure 1, Table 1). Both families include additional genera of non-methanotrophic bacteria. Nearly all methanotrophic *Gammaproteobacteria* are classified into the families *Methylococcaceae* or the recently delineated *Methylothermaceae* (Hirayama et al., 2014). These families do not contain any non-methanotrophic bacteria. “*Candidatus* Crenothrix polyspora” is the only exception as it belongs to a distinct family, the *Crenotrichaceae*, but this classification was put into question by Op den Camp et al. (2009), who proposed that *Crenothrix* could be a member of the *Methylococcaceae*, based on its 16S rRNA gene phylogeny (Figure 1).

### Classification of cultivated methanotrophic bacteria into type I and type II methanotrophs?

The characterization of several new genera of methanotrophs in the 1970s and 1980s resulted in the classification into two major groups, type I and type II methanotrophs based on physiological, morphological, ultrastructural and chemotaxonomic traits (Whittenbury and Dalton, 1981). Major distinctive characteristics between type I and type II methanotrophs were the arrangement of internal membranes as vesicular discs (type I) or paired membranes aligned to the cell periphery (type II), the carbon fixation mechanism via the ribulose monophosphate pathway (type I) or serine cycle (type II), the capability of nitrogen fixation, the formation of resting stages, and the predominance of specific C_16_ (type I) or C_18_ (type II) fatty acids (Hanson and Hanson, 1996; Trotsenko and Murrell, 2008). In some studies, type X methanotrophs were further differentiated from type I methanotrophs based on characteristics such as the presence of ribulose-1,5-bisphosphate carboxylase, differences in nitrogen fixation capability or higher optimum growth temperatures (Green, 1992; Hanson and Hanson, 1996; Bowman, 2006). Phylogenetic analyses of 16S rRNA gene sequences confirmed this classification, whereby type I and type X methanotrophs correspond to the *Gammaproteobacteria* and type II to the *Alphaproteobacteria*. However, the characterization of several new genera and species during the last years has turned this distinction based on the mentioned criteria largely into question. While the major carbon fixation pathway is still a distinctive feature, several other characteristics are no longer exclusively found in one or the other group:

1. Methanotrophic *Beijerinckia* species are not considered as typical type II methanotrophs as most of them lack the characteristic internal membrane system. Some may have vesicles instead, but only *Methylocapsa palsarum* has a well-developed membrane system. Furthermore, the genera *Methylocella* and *Methyloferula* do not possess the particulate methane monooxygenase (Dedysh et al., 2000, 2002, 2004, 2015a; Dunfield et al., 2003, 2010; Vorobev et al., 2011).
2. All methanotrophic *Beijerinckia* species lack the classical 18:1ω8c signature fatty acid of type II methanotrophs (Dedysh et al., 2000, 2002, 2004, 2015a; Dunfield et al., 2003, 2010; Vorobev et al., 2011). Similarly, *Methylosinus sporium* does not possess this signature fatty acid (Bodelier et al., 2009).
3. *Methylocystis heyeri* possesses with 16:1ω8c a signature fatty acid of type I methanotrophs (Dedysh et al., 2007).
4. Most members of the *Methylothermaceae* have signature fatty acids of type II methanotrophs: *Methylohalobius crimeensis, Methylothermus subterraneaus*, and *Methylomarinovum caldicuralii* contain 18:1ω7c among their major fatty acids (Heyer et al., 2005; Hirayama et al., 2010, 2014). The fourth member of this family, *Methylothermus thermalis*, contains 18:1ω9c, a C_18_ fatty acid neither abundant in the other *Methylothermaceae* nor in type II methanotrophs (Tsubota et al., 2005).
5. Methanotrophic *Verrucomicrobia* do not fit well into the scheme as most of them lack the typical intracytoplasmic membranes (only exception “*Methylacidimicrobium fagopyrum*”) and have distinct dominant fatty acids (i14:0, a15:0) (Op den Camp et al., 2009; van Teeseling et al., 2014).
6. Further differentiation criteria such as nitrogen fixation capability, the formation of resting stages, or the optimum growth temperature, which were initially applied, are not indicative for one or the other type anymore.

Based on these exceptions, the initial concept of type I and II methanotrophs is no longer useful to categorize all known aerobic methanotrophic bacteria and it has been proposed to abandon it (Op den Camp et al., 2009; Semrau et al., 2010). Nevertheless, the terms are still frequently used and adapted to the increasing diversity of methanotrophs, but should only be considered as synonyms for the phylogenetic groups of methanotrophic *Alpha*- and *Gammaproteobacteria*. In this way the terms will be used in this review.

The methanotrophic *Alphaproteobacteria* were recently divided into type IIa (*Methylocystaceae*) and type IIb (*Beijerinckiaceae*) methanotrophs (Deng et al., 2013; Dumont et al., 2014). Likewise, the methanotrophic *Gammaproteobacteria* are frequently differentiated into subgroups. Often they are divided into two groups, whereby the genera *Methylococcus, Methylocaldum, Methylogaea* and the *Methylothermaceae* form type 1b methanotrophs, while the remaining gammaproteobacterial genera are grouped as type 1a methanotrophs (Chen et al., 2008; Deutzmann et al., 2011; Dumont et al., 2011; Siljanen et al., 2011; Krause et al., 2014). Some recent studies differentiated the methanotrophic *Gammaproteobacteria* into three type I subgroups, but this categorization is not consistent among different publications. A taxonomic review referred to the clade consisting of *Methylococcus, Methylocaldum, Methylogaea, Methyloparacoccus* as type Ia and to members of the family *Methylothermaceae* as type Ic, while the remaining *Gammaproteobacteria* represented type Ib methanotrophs (Bowman, 2014). In contrast, in some cultivation independent studies the above mentioned frequent grouping into type Ia and Ib was applied and extended by introducing type Ic, comprising *pmoA* sequences of uncultivated taxa (USCγ, JR2, JR3, OPU1) and the *amoA* sequence of *Nitrosococcus* (Lüke and Frenzel, 2011; Henneberger et al., 2012; Dumont et al., 2014). It is thus referring to a group of uncultivated methanotrophs. Such a further differentiation of the methanotrophic *Gammaproteobacteria* appears useful to refer to the specific subgroups of cultivated and uncultivated methanotrophs easily. In this review, the nomenclature of type Ia and Ib methanotrophs as applied in diverse cultivation-independent studies is kept, while the *Methylothermaceae* are referred to as type Ic methanotrophs (Table 2). The clade of *Nitrosococcus* and related uncultivated clusters represent type Id organisms when discussing diversity based on *pmoA* phylogeny. Methanotrophic *Verrucomicrobia* are referred to as type III.

### Ecophysiology of aerobic methanotrophic bacteria

Aerobic methanotrophic bacteria occur in terrestrial, aquatic and marine ecosystems, typically at oxic-/anoxic interfaces, where oxygen is available as electron acceptor and methane as carbon and energy source, which is released as end product from the anaerobic degradation of organic matter. They are likewise present in diverse upland soils where they are responsible for atmospheric methane oxidation or become temporarily active when higher concentrations of methane are available (Knief et al., 2006; Dunfield, 2007; Kolb, 2009). The ecology of methanotrophic bacteria has been reviewed in diverse articles and will not be discussed in detail here (e.g., Hanson and Hanson, 1996; Conrad, 2007; Semrau et al., 2010; Chowdhury and Dick, 2013; Bowman, 2014). The focus in this article is on physiological adaptations to particular environmental conditions in relation to phylogeny.

In terms of metabolic adaptations, some methanotrophic bacteria show higher versatility than initially thought. They are capable of growing on carbon compounds with C-C bond, while most methanotrophic bacteria are obligate methanotrophs. The existence of such facultative methanotrophs had been debated for a long time (reviewed in Theisen and Murrell, 2005; Semrau et al., 2011), until it was rigorously proven for *Methylocella silvestris* BL2 (Dedysh et al., 2005). This strain has the broadest versatility currently known among methanotrophs; besides C_1_-compounds, it can use a variety of organic acids including acetate, pyruvate, propionate, succinate, malate, and gluconate, alcohols such as ethanol and 2-propanol and the gaseous compounds ethane and propane (Crombie and Murrell, 2014). Growth on acetate is more efficient than on methane and methane monooxygenase expression is down-regulated in the presence of acetate (Dedysh et al., 2005; Theisen et al., 2005). In contrast, methane and propane are consumed simultaneously in this strain (Crombie and Murrell, 2014). A facultative lifestyle with a much narrower substrate range has been reported for other members of the genus *Methylocella* and for *Methylocapsa aurea* (Table 1), but it is not a general feature of all methanotrophic *Beijerinckiaceae*. Moreover, several *Methylocystis* strains including diverse type strains are able to grow on acetate or ethanol, but with growth rates 3–10-fold lower compared to growth on methane (Belova et al., 2011; Im et al., 2011). Gene expression of methane monooxygenase appears not to be regulated by acetate in these methanotrophs (Belova et al., 2011; Yoon et al., 2011). It remains to be proven whether the capability to grow on acetate is linked to phylogeny within this genus. *Crenothrix polyspora* is the only methanotrophic gammaproteobacterium for which uptake of acetate and, to lesser extent, glucose, has been reported (Stoecker et al., 2006), but besides evidence from fluorescence *in situ* hybridization experiments coupled to microautoradiography (FISH-MAR) performed on natural enrichments, this phenomenon has not been further proven. It is obvious that a facultative lifestyle can provide a benefit for methanotrophic bacteria. However, the relevance of facultative methanotrophy in nature remains little understood, and linked to this the question to what extent a facultative life style may influence methane emissions in the environment. Only few studies have analyzed the consumption of methane and alternative substrates under *in situ* conditions so far. In mire samples, acetate addition resulted in a reduction of methane emission rates and decreased *pmoA* expression rates of *Methylocystis* (Wieczorek et al., 2011). Likewise, acetate addition decreased methane oxidation rates and stimulated growth of *Methylocystis* in paddy soil samples. Stable isotope probing with ^13^C-labeled acetate under aerobic conditions resulted in a labeling of *Methylocystis* in these samples, demonstrating that the labeled carbon was somehow metabolized and incorporated by the cells (Leng et al., 2015).

Another aspect that has repeatedly been addressed is the adaptation to low methane concentrations. The observations made in competition experiments with isolates grown in continuous culture and in incubations with rice field soils resulted in the frequently cited conclusion that type I methanotrophs are more competitive under low methane concentrations compared to type II methanotrophs (Graham et al., 1993; Henckel et al., 2000b; Macalady et al., 2002). This seems to apply to ecosystems as long as methane supply remains at a rather high level, but when methane concentrations drop below 1000 or even 100 ppmv for prolonged periods of time, *Methylocystaceae* have the better potential to remain active (Knief and Dunfield, 2005).

Most methanotrophic bacteria are mesophilic and neutrophilic organisms, but several isolates were obtained from more extreme habitats and are specifically adapted to lower or higher pH, temperature, salt or oxygen concentrations (Trotsenko and Khmelenina, 2002). Methanotrophic bacteria adapted to warmer or colder temperatures are found in a couple of distinct genera of *Gammaproteobacteria*, often side by side with mesophilic species (Table 2). Among the methanotrophic *Alphaproteobacteria* adaptations to temperatures outside the mesophilic range appear to be uncommon. Outstanding are the verrucomicrobial methanotrophs, which represent the most thermophilic methanotrophs (optimum temperature 55–60°C) (Op den Camp et al., 2009). These are at the same time acidophiles, with pH optima for growth between 2.0 and 4.3. All isolates were obtained from geothermally influenced environments (Op den Camp et al., 2009; van Teeseling et al., 2014). The occurrence of these thermoacidophilic methanotrophs appears to be largely restricted to such geothermal environments, in particular to acidic conditions, while they seem to have a broader temperature range, as revealed by cultivation-dependent and -independent analyses (Sharp et al., 2014; van Teeseling et al., 2014).

An adaptation to mildly acidic pH values (growth optima between 5.0 and 6.0) is characteristic for methanotrophic *Beijerinckiaceae* and some *Methylocystis* strains, which were mostly isolated from acidic peatlands or forest soils (Table 1). Cultivation-independent analyses suggest that the occurrence of *Methylocella* is not limited to these acidic environments (Rahman et al., 2011). Less common are acidophilic methanotrophs among the *Gammaproteobacteria*. Members of the species *Methylomonas paludis* have been described as acid-tolerant and are inhabitants of acidic peatlands (Danilova et al., 2013; Danilova and Dedysh, 2014). Methanotrophs that are adapted to high pH values are found within the *Gammaproteobacteria*, in particular within the genus *Methylomicrobium*. The occurrence of alkaliphilic methanotrophs is not restricted to the class of *Gammaproteobacteria*, the isolation of an alkaliphilic *Methylocystis* isolate has also been reported (Eshinimaev et al., 2008). Some alkaliphilic *Gammaproteobacteria* are at the same time halophiles (*Methylomicrobium alcaliphium* and *Methylomicrobium kenyense*), isolated from soda lakes (Kalyuzhnaya et al., 2008). Methanotrophic bacteria that were isolated from marine ecosystems are also adapted to higher salt concentrations and are likewise found among methanotrophic *Gammaproteobacteria*. High salt tolerance is not necessarily a characteristic of all members of a genus, as exemplified by *Methylocaldum* and *Methylomicrobium* (Table 2). In the last few years, the first methanotrophic isolates were described that live preferentially under lower oxygen concentrations (*Methylosoma difficile* and *Methyloglobulus morosus*). They were enriched in systems with opposing gradients of methane and oxygen, thus mimicking the conditions in sediments (Rahalkar et al., 2007; Deutzmann et al., 2014).

In conclusion, a broad versatility in terms of adaptation to different environmental conditions can be found among the methanotrophic *Gammaproteobacteria* (low and high temperatures, low and high pH, high salt, low oxygen), which comes along with a high diversity of methanotrophs within this group. Cultivated methanotrophic *Alphaproteobacteria* are less diverse and show less and different adaptations (low pH, low methane availability) based on current knowledge. At genus level, the occurrence of methanotrophic bacteria that are adapted to a specific environmental condition is not necessarily limited to one phylogenetic lineage, but can often be found within different genera of methanotrophs side by side with species that show different adaptations and habitat preferences. Thus, some genera have a broad ecological niche, though the individual species or strains have smaller niches, while others are less diverse in term of ecophysiological adaptations and have a rather narrow niche. Habitat adaptation and specialization appear to occur at different taxonomic levels. Consequently, the distribution of methanotrophic bacteria in the environment should be evaluated at these different taxonomic levels in order to better understand distribution and community composition. Such a detailed evaluation is undertaken in this review, based on a meta-analysis including the large diversity of uncultivated methanotrophs (see Sections Description of Major Uncultivated Groups of Methanotrophic Bacteria and Their Habitat Specificity and Habitat Specificity of Methanotrophic Taxa Evaluated at Higher Taxonomic Resolution).

## Cultivation-independent detection of aerobic methanotrophic bacteria based on molecular markers

Tools for the cultivation-independent detection of aerobic methanotrophic bacteria exist since 20 years and have been used in diverse studies. The most frequently targeted gene in environmental studies, the 16S rRNA gene, can be used for the detection of methanotrophic bacteria using taxon specific primers and probes that are available for several different groups (compiled by McDonald et al., 2008). While the analysis of this gene provides valuable information about the phylogenetic placement of methanotrophic bacteria detected in environmental samples, it does not allow the identification of methanotrophic bacteria beyond the well-known families.

### Functional marker genes as molecular markers

Such a limitation is of less relevance when functional genes are used as markers, such as the methane monooxygenase encoding genes (McDonald et al., 2008). The methane monooxygenase is the key enzyme responsible for the initial conversion step of methane to methanol. Two forms of this enzyme are known, the soluble methane monooxygenase (sMMO) and a membrane-bound enzyme, the particulate methane monooxygenase (pMMO). The *pmoA* gene encoding the β-subunit of the particulate methane monooxygenase is the most frequently used marker, as it is present in most aerobic methanotrophic bacteria with exceptions among the *Beijerinckiaceae* (Table 1). It is also present in anaerobic denitrifying bacteria, represented by an enriched culture of “*Candidatus* Methylomirabilis,” a bacterium of the NC10 phylum (Ettwig et al., 2010).

To include *Beijerinckiaceae* and to obtain a more complete picture about the methanotrophs present in a sample, the *mmoX* gene encoding the α-subunit of the soluble methane monooxygenase hydroxylase component has been used in addition to *pmoA* in some studies (e.g., Morris et al., 2002; Chen et al., 2008; Deng et al., 2013). However, due to its limited occurrence in methanotrophs (Tables 1–3), *mmoX* is much less frequently used as marker. It is not uniformly present or absent within the same genus and variation exists even at species level, as evident from studies with *Methylocystis, Methylosinus*, and *Methylomonas* strains (Shigematsu et al., 1999; Heyer et al., 2002).

Further gene markers that can be used for the detection of methanotrophs are not unique to this metabolic guild, but shared with other organisms. Among those are the *mxaF* gene, which encodes the large subunit of the methanol dehydrogenase, and a couple of other markers of the methylotrophic metabolism (reviewed by Kolb and Stacheter, 2013; Dumont, 2014).

### *pmoA* as molecular marker

Both, *pmoA* and *mmoX* have been shown to produce phylogenies that are largely congruent with those of the 16S rRNA gene (Auman and Lidstrom, 2002; Heyer et al., 2002; Kolb et al., 2003), which allows to draw conclusions about the phylogenetic placement of methanotrophs possessing genes with novel sequence types. Updated trees (Figure 1) show that this is still the case, but research of the last few years has revealed that this congruency includes more and more exceptions. The presence of paralogous gene copies in methanotrophic bacteria as well as the detection of evolutionary related monooxygenases in non-methanotrophic bacteria contribute to sequence diversity in cultivation-independent studies (see next section). Hence, conclusions about the taxonomic identity of bacteria detected based on their *pmoA* sequences have to be drawn with care, in particular if sequences cluster distantly to those of well-known methanotrophs. This is also exemplified by the *pmoA* sequence of the gammaproteobacterial “*Candidatus* Crenothrix polyspora,” which is highly divergent from those of all other methanotrophic *Gammaproteobacteria*. Besides these issues, inconsistency exists among the type Ia methanotrophs (Figure 1). Tree reconstructions within this group are in general not highly robust, but both, *Methylobacter* and *Methylomicrobium* species do not form monophyletic clusters, independent of the applied treeing method and the phylogenetic marker. *Methylobacter psychrophilus* and *Methylobacter tundripaludum* appear to be distinct from the other *Methylobacter* species, likewise as *Methylomicrobium album* and *Methylomicrobium agile* cluster with *Methylobacter whittenburyi* in 16S rRNA gene based trees and with *Methylosarcina* species in *pmoA* based trees rather than with the other *Methylomicrobium* species. Elaborate taxonomic analyses including information derived from whole genome sequencing projects of these and further reference strains will be necessary to ensure the taxonomic placement of these species.

A couple of different primer sets were developed for the amplification of *pmoA* gene fragments, but remarkably, the first published primer pair (A189/A682) is still most frequently used (Holmes et al., 1995). Only one alternative system (A189/mb661) is often used instead or in addition to the before mentioned system (Costello and Lidstrom, 1999). This second primer combination is more specific for methanotrophic bacteria as it does not amplify the *amoA* gene of ammonia-oxidizing bacteria (Costello and Lidstrom, 1999). However, it fails to detect some of the clusters that have a phylogenetic position between *pmoA* and *amoA* sequences, such as the RA21 or the *pxmA* cluster, it largely discriminates USCα and amplifies type IIb methanotrophs less efficiently (Bourne et al., 2001; Deng et al., 2013). A third primer, A650 does not show this limitation while excluding *amoA*, but has less frequently been used (Bourne et al., 2001; Shrestha et al., 2012). Because primer system A189/A682 results in the production of additional unspecific PCR products in some cases, a semi-nested approach was used in these studies. After a first PCR using primers A189/A682 a second PCR with primers A189/mb661 or A189/A650 was applied (Singh et al., 2007; Qiu et al., 2008; Kip et al., 2011; Siljanen et al., 2011; Barbier et al., 2012). Alternatively, a combination of both reverse primers in a multiplex PCR was used in the second PCR to overcome the detection limitations of primer mb661 (Horz et al., 2005). Some further general and several specific primers for the detection of subgroups were developed, as compiled in review articles (McDonald et al., 2008; Dumont, 2014). Many of them were developed for qPCR assays targeting subgroups (Kolb et al., 2003, 2005; Degelmann et al., 2010; Wieczorek et al., 2011; Sharp et al., 2014). Moreover, specific primers are needed to amplify *pmoA* genes of *Verrucomicrobia* (Erikstad et al., 2012; Sharp et al., 2012), the homologous *pmoA2* gene (Tchawa Yimga et al., 2003), or the *pmoA* genes of anaerobic methanotrophic bacteria of the NC10 phylum (Luesken et al., 2011).

## *pmoA* paralogs and evolutionary related monooxygenases

Paralogous copies of the *pmoA* gene and evolutionary related monooxygenases in non-methanotrophic bacteria are sometimes detected in cultivation-independent studies, depending on the primers used to amplify the target gene. They can thus contribute to the diversity of detected sequence types in environmental studies, but do not represent distinct methanotrophs. A couple of sequence clusters in *pmoA* based phylogenetic trees have meanwhile been identified as paralogs or alternative monooxygenases.

### *pmoA* paralogs in methanotrophic bacteria

Many methanotrophs have multiple copies of the *pmo* operon and initially it appeared that these copies are (nearly) identical (Auman et al., 2000; Gilbert et al., 2000), so that they do not affect diversity studies that are based on *pmoA* gene detection. *Methylocystis* sp. SC2 was the first methanotrophic strain in which two different *pmoA* genes were discovered, the conventional and a second copy, referred to as *pmoA2*, with only 73% identity to the well-known *pmoA* gene of *Methylocystaceae* (Dunfield et al., 2002). The application of specific primers for the detection of the *pmoA2* gene revealed that this second gene copy is present in diverse though not all *Methylocystis* and *Methylosinus* strains (Tchawa Yimga et al., 2003). The *pmoA2* gene is localized in the *pmoCAB2* operon, which encodes a functional methane monooxygenase, enabling *Methylocystis* SC2 to oxidize methane at lower mixing ratios compared to the conventional monooxygenase, which is downregulated under these conditions in strain SC2 (Baani and Liesack, 2008). This finding was taken as explanation for the previously described capability of *Methylocystis* species to oxidize methane at very low mixing ratios down to atmospheric level over a period of several months and their capability to grow at mixing rations as low as 10–100 ppmv (Knief and Dunfield, 2005). Moreover, this corresponds very well to the observation that *Methylocystis* strains are frequently detected in upland soils and hydromorphic soils, where they face low methane supply almost constantly (Dunfield, 2007). However, the *pmoA2* gene of *Methylocystis* and *Methylosinus* has not been detected very frequently in upland soils, but rather in different other ecosystems (Tables S1–S4). Either the commonly applied primers are not well suited to amplify *pmoA2* genes of those *Methylocystaceae* that occur in upland soils, or the *pmoA2* gene is more important for survival of methanotrophs residing in habitats with fluctuating methane supply at higher concentrations.

In methanotrophic *Verrucomicrobia*, multiple different *pmoA* gene copies are present (Figure 1). All genes are highly divergent from those of proteobacterial methanotrophs and quite different to each other (Op den Camp et al., 2009). The strains “*Methylacidiphilum fumariolicum”* SolV and “*Methylacidiphilum infernorum”* V4 possess three complete *pmoCAB* operons, while “*Methylacidiphilum kamchatkense”* Kam1 has a fourth distinct copy of *pmoA*, localized in a truncated *pmoCA* operon. An expression study performed with this strain revealed that the methane monooxygenase encoded by *pmoCAB2* is strongly expressed when cells are grown under laboratory conditions (Erikstad et al., 2012). The function of the other copies and regulatory mechanisms that may control the expression of these genes remain currently largely unknown.

### The *pxmA* gene

In methanotrophic *Gammaproteobacteria* of the genera *Methylomonas, Methylobacter* and *Methylomicrobium* another homolog of *pmoA* has been detected, the *pxmA* gene (Tavormina et al., 2011). Recent genome sequencing projects reveal that *pxmA* genes occur more widespread in methanotrophs. They are present in further *Methylococcaceae* strains, which are distantly related to the known genera but have so far not been further described in the literature. A *pxmA* copy is also present in an alphaproteobacterial strain, *Methylocystis rosea*. In *Methyloglobulus morosus* an additional *pxmA* like gene is present besides *pmoA* and *pxmA*. All *pxmA* gene sequences form a monophyletic cluster that is clearly distinct from *pmoA* sequences of methanotrophic *Proteobacteria* and *Verrucomicrobia* (Figure 1). Already before their description by Tavormina et al. (2011), *pxmA* genes were detected in environmental samples, they were referred to as “*pmoA/amoA* like” sequences or as Cluster WC306-54 (Nold et al., 2000; Lau et al., 2007; Dörr et al., 2010). The presence of *pxmA* appears not to be closely linked to phylogeny, similarly to the occurrence of *pmoA2* among *Methylocystaceae* or *mmoX* among the methanotrophic Proteobacteria. The function of the gene product and regulation of gene expression remain currently largely unknown. So far, it could be shown that the gene, which is localized in the *pxmABC* operon, is expressed under environmental and *in vitro* conditions (Tavormina et al., 2011; Kits et al., 2015).

### Evolutionary related monooxygenases

It is well known that the particulate methane monooxygenase and the ammonia monooxygenase of nitrifying bacteria and archaea are evolutionary related (Holmes et al., 1995). Meanwhile, further monooxygenases of the superfamily of copper-containing membrane-bound monooxygenases have been identified, involved in the oxidation of short chain hydrocarbons, but not methane (Redmond et al., 2010; Sayavedra-Soto et al., 2011; Coleman et al., 2012; Suzuki et al., 2012). In phylogenetic trees, the sequences of these genes form clusters that are distantly related to those of the known *pmoA* and *amoA* genes. Due to the high sequence divergence, most of these sequence types have not frequently been detected in cultivation-independent PCR-based studies using current *pmoA* primers, but some of them have been found in metagenomic or metatranscriptomic datasets, e.g., in hydrocarbon-rich marine ecosystems (Li et al., 2014).

The existence of a butane monooxygenase in *Nocardioides* sp. CF8 related to the particulate methane monooxygenase was already postulated by Hamamura and Arp (2000), but molecular evidence was provided only recently when the whole genome of the strain was sequenced (Sayavedra-Soto et al., 2011). The butane-oxidizing monooxygenase is encoded by the genes in the *bmoCAB* operon, which have less than 50% amino acid similarity to the genes of the methane and ammonia monooxygenase. Similar genes were also detected in *Mycobacterium smegmatis* strains NBB4 and NBB3 (Coleman et al., 2012). The enzyme in strain NBB4 was shown to oxidize ethane, propane, butane and ethylene. Due to the broader substrate spectrum of the enzyme in *Mycobacterium*, the enzyme was referred to as hydrocarbon monooxygenase, encoded in the *hmoCAB* operon. Genome sequencing projects suggest that similar monooxygenases exist in *Mycobacterium chubuense* B4, *Nocardioides luteus* FB or the uncultured deltaproteobacterial SAR324 clade, which is ubiquitous in the ocean (Sheik et al., 2014).

Redmond et al. (2010) described another cluster of putative hydrocarbon monooxygenases (*emoA*), detected upon stable isotope probing with ^13^C-ethane at a hydrocarbon seep. The authors speculate that the labeled organisms are members of the *Methylococcaceae*, which seem to be incapable of methane oxidation. These assumptions can currently only be confirmed by sequence data from isolates referred to as *Methylococcaceae* ET-SHO and ET-HIRO, which were deposited in the NCBI database in an independent study, but remain to be published. Based on the entries in the NCBI database it appears that these *Methylococcaceae* isolates, which were also obtained from a marine habitat, could grow on ethane, but not on methane.

Further types of monooxygenase genes related to *pmoA* and *amoA* are found in *Gammaproteobacteria* of the genus *Haliea* and in the genome of the alphaproteobacterium *Skermanella aerolata* KACC 11604 (= 5416T-32^T^). Strains *Haliea* ETY-M and ETY-NAG grow on ethylene and oxidize in addition ethane, propane and propylene, but not methane (Suzuki et al., 2012). In case of *Skermanella aerolata* KACC 11604 growth on hydrocarbons has not yet been studied. The sequence of their monooxygenase is different from the *hmoA* and *emoA* genes, but related to the *pmoA* sequences of type II methanotrophs.

## A comparison of cultivation-dependent and –independent diversity of methanotrophs based on *pmoA* as phylogenetic marker

### Classification of *pmoA* sequences based on phylotyping or OTU clustering

Using *pmoA* as molecular marker for the detection of methanotrophic bacteria it turned out that there is a huge diversity of methanotrophs present in nature that is not represented by isolates in the laboratory. Approximately 15,000 *pmoA* and *pmoA*-like sequences can be found in the Genbank database. To describe and discuss the current diversity of aerobic methanotrophic bacteria based on this data resource, sequences have to be grouped based on similarity. In many studies such groups are defined based on their clustering in phylogenetic trees in relation to known phylotypes, which are represented by sequences of type strains or other well-studied reference strains as well as selected sequences of uncultivated clades. Dumont et al. (2014) recently defined 53 representative sequences for major cultivated and uncultivated phylogenetic clusters.

**Figure 2:**
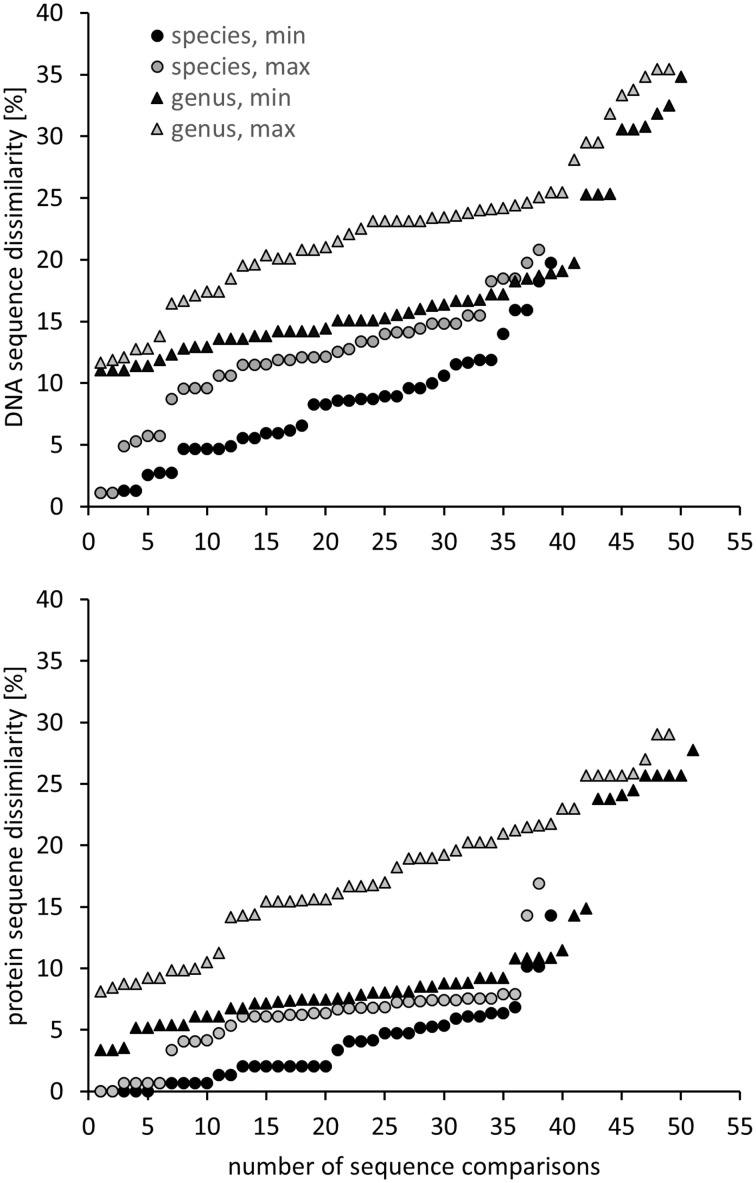
**Minimum and maximum ***pmoA*** sequence dissimilarity at DNA (upper panel) and protein level (lower panel) between a type strain and its most closely and most distantly related type strain within the same species, as well as to the most closely and distantly related type strain of a different genus within the same family or type (according to Table 1)**. DNA and protein distance matrices were calculated in ARB based on 480 aligned nucleotide positions or 160 deduced amino acid positions. *Methylomicrobium album* and *Methylomicrobium agile* were not included, due to the very distant clustering from the other *Methylomicrobium* strains (Figure 1), while “*Candidatus* Crenothrix polyspora” was excluded due to the fact that it contains a highly divergent *pmoA* sequence compared to all other *Gammaproteobacteria*.

Another approach is the grouping of similar sequences into operational taxonomic units (OTUs) using a predefined cut-off value. Some studies applied a 3% cut-off without explicitly linking this to a specific phylogenetic resolution (Saidi-Mehrabad et al., 2013; Sharp et al., 2014). Other studies determined and used cut-off values with the aim to reflect genus and species resolution. These values were determined in correspondence to the routinely used cut-off values known from 16S rRNA gene sequence analyses, i.e., 3% sequence difference to distinguish between species and 5% to differentiate genera (Schloss and Handelsman, 2005). For *pmoA* sequences, Lüke et al. (2010) defined cut-off values at 10 and 17% sequence dissimilarity for species and genus delineation, respectively, based on the fact that the nucleotide substitution rate of *pmoA* is 3.5 times higher than that of 16S rRNA genes. The factor 3.5 was derived by correlation of 16S rRNA and *pmoA* gene sequence identities of approx. 75 *Methylocystis* and *Methylosinus* strains (Heyer et al., 2002). Degelmann et al. (2010) included *Gammaproteobacteria* in the comparative analysis and compiled 16S gene sequence identity values of 22 methanotrophs. They correlated 16S rRNA gene to *pmoA* gene as well as to deduced PmoA protein sequence identity values and defined a cut-off of 13% at DNA level for species delineation, corresponding to 7% cut-off at protein level. When comparing these cut-off values to the sequence differences observed between methanotrophic type strains within the same and of different genera, it is apparent that they reflect the average sequence difference between type strains so that genera and species will not be fully resolved using these values (Figure 2). At the same time the diagrams, which display minimum and maximum sequence difference of each type strain to another type strain within the same genus and family, reveal that it will be impossible to find cut-off values that differentiate perfectly well all genera without already differentiating species within a genus. Similar difficulties in determining cut-off values that correspond to a certain phylogenetic resolution are known from 16S rRNA gene based analyses (Schloss and Westcott, 2011).

**Figure 3:**
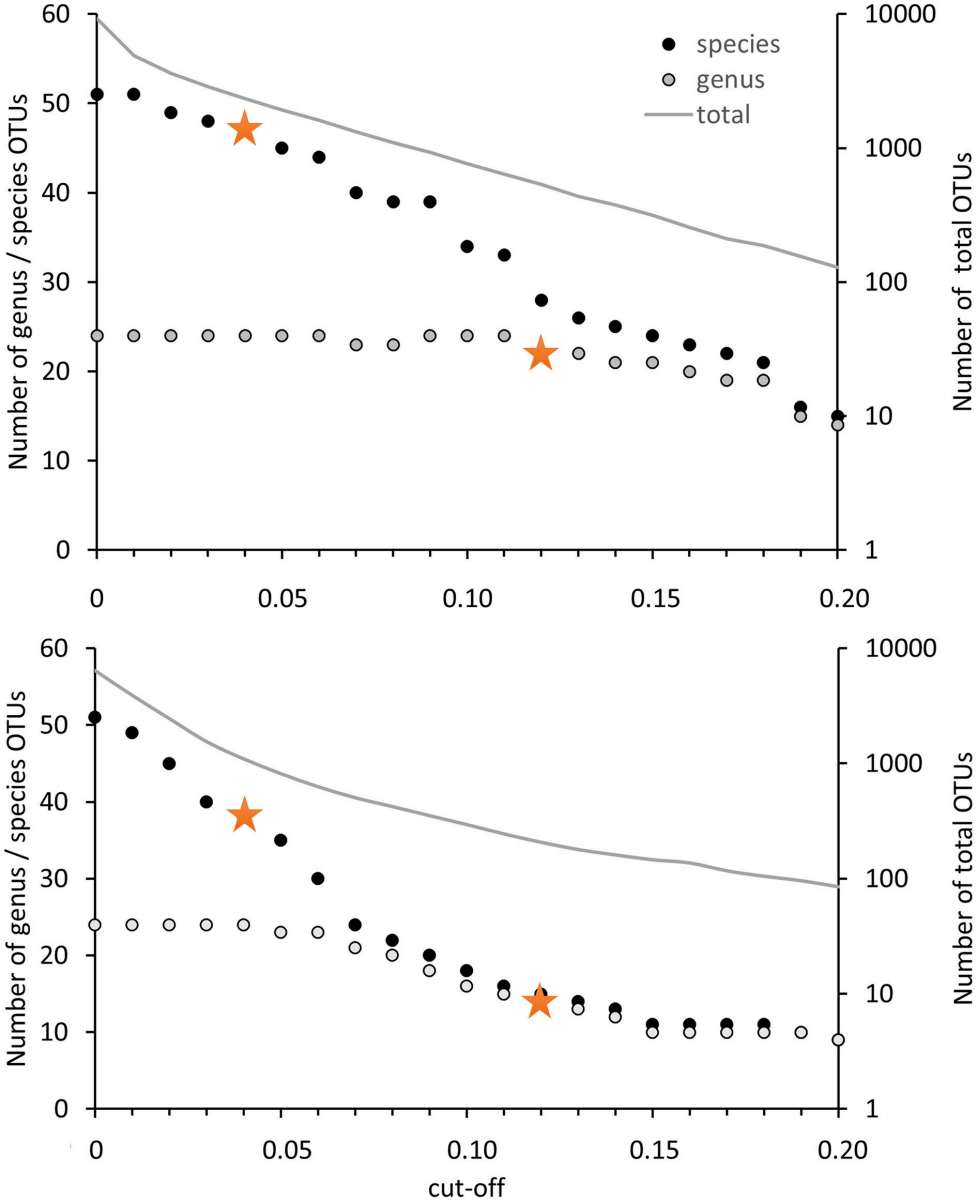
**Number of OTUs in dependence on the cut-off value applied for OTU differentiation**. The number of OTUs containing type strains of different genera or species are displayed on the left axis, the number of OTUs formed based on all high quality sequences (= total) is presented on the right axis at logarithmic scale. Clustering was performed with 12502 high quality *pmoA* sequences (upper panel) or the deduced amino acid sequences (lower panel) available from Genbank. Sequences with at least 400 bp sequence length and without accumulation of sequencing errors were included. Distance matrices were calculated in ARB based on 480 aligned nucleotide positions or 160 deduced amino acid positions. OTU clustering was done using Mothur by applying the average neighbor algorithm. Orange stars denote the cut-off values applied in this review.

For the evaluation of the diversity of methanotrophic bacteria in this review article, OTU clustering was performed based on cut-off values that reflect a higher resolution compared to the published values to resolve the distinct genera and species as good as possible. The compilation of minimal DNA sequence differences between genera reveals that a cut-off value of 11% is necessary to differentiate all genera (Figure 2). Indicative for an adequate resolution is the separation of the two most closely related genera, *Methylocystis* and *Methylosinus*. To further evaluate the 11% cut-off value, it was applied to cluster all available high quality *pmoA* sequences using the Mothur classification tool with average neighbor algorithm. Sequences of at least 400 bp length and without accumulation of evident sequencing errors were considered as high quality here and the dataset is referred to as “large *pmoA* dataset” in the following. When performing OTU clustering using different cut-off values it turned out that not 11% but 12% cut-off is sufficient for nearly full resolution at genus level (Figure 3). At the same time, type strains belonging to the same genus were grouped into distinct clusters in five cases: “*Methylacidiphilum*,” “*Methylacidimicrobium,” Methylocapsa, Methylomicrobium*, and *Methylobacter*. In case of *Methylobacter* and *Methylomicrobium*, this finding corresponds to the polyphyletic clustering in *pmoA* trees (Figure 1). To fully prevent the formation of more than one OTU for these genera, a much higher cut-off value of >20% would be necessary.

The differentiation of *pmoA s*equences at species level is affected by similar difficulties. A cut-off value of 1% is necessary to resolve all species (except *Methylomicrobium album* and *Methylomicrobium agile*, which have even more similar *pmoA* sequences), while such a low value will classify at the same time many strains belonging to the same species into distinct taxonomic units. A higher cut-off value of 3 or 4% leaves only some species unresolved (Figure 2), namely the two *Methylothermus* species, *Methylocystis hirsuta*, and *Methylocystis rosea*, as well as some of the *Methylomicrobium* species. OTU clustering applied to the “large *pmoA* dataset” confirmed these findings and shows that a cut-off value of 4% is sufficient to differentiate the majority of species.

Phylogenetic analysis of functional genes is frequently based on protein sequences. This excludes sequence variability at nucleotide positions that are not under evolutionary selection pressure, but provides at the same time less information, so that resolution of closely related taxa becomes more difficult. OTU clustering of sequences with a cut-off that roughly reflects genus level resolution can be achieved at 6% sequence dissimilarity (Figure 2). It only fails to resolve *Methylomarinovum* from *Methylothermus*, but a lower value should nevertheless not be selected as the 6% value already provides higher resolution compared to the 12% cut-off value at DNA level when considering the large PmoA dataset including sequences of uncultivated methanotrophs (Figure 3). Differentiation of species based on protein sequences is even more difficult. Full resolution cannot be obtained as *Methylomicrobium* and *Methylothermus* species are not even separated at 1% cut-off. A cut-off of 2% already fails to resolve the majority of type strains within the genera *Methylocystis, Methylomicrobium*, and *Methylothermus*, although it still gives a higher number of OTUs compared to the 4% cut-off at DNA level when sequences from cultivation-independent studies are included (Figure 3).

Due to the difficulties in finding appropriate cut-off values at protein level, *pmoA* sequence diversity was evaluated based on DNA sequences but not protein sequences in the present work. The 12% cut-off was applied to differentiate sequences at a level that allows resolution of most methanotrophic genera and a 4% cut-off was used to differentiate species reasonably well. To distinguish in the following OTU classification done with 12% cut-off from classification with 4% cut-off, the OTUs are referred to as OTU_12_ and OTU_4_, respectively.

### How well do cultivated strains cover the diversity of methanotrophic bacteria as seen based on cultivation-independent studies?

Of the 15,000 *pmoA* sequences that have been deposited in the Genbank database, the vast majority was derived from cultivation-independent studies. Slightly less than 3% were obtained from cultured methanotrophic strains. Most of them belong to the well-known genera *Methylocystis, Methylosinus, Methylomonas, Methylobacter, Methylocaldum*, or *Methylomicrobium* (Table 4). Approximately 20 sequences represent isolates that cannot be assigned to a specific known genus; at least some of them may represent new genera. At species level, isolates that are similar to *Methylocystis rosea, Methylocystis hirsuta, Methylocystis echinoides, Methylosinus sporium*, and *Methylosinus trichosporium* or “*Methylomonas denitrificans*” have most frequently been obtained (Table 5). In contrast, more than half of the described species are represented by only one single strain at the moment.

**Table 4:** **Detection frequency of methanotrophic genera in cultivation-dependent and -independent studies**.

**Genus**	**Number of reads from isolates**	**Reads in OTUs_12_**	**Reads in OTUs_4_**
*Methylocystis*	141	2754	1743
*Methylosinus*	95	173	141
*Methylomonas*	43	690	98
*Methylobacter*	34	743	153
*Methylocaldum*	16	283	254
*Methylomicrobium*	13	67	78
“*Methylacidiphilum”*	10	10	10
*Methylococcus*	7	320	282
“*Candidatus* Crenothrix”	6	43	9
“*Methylacidimicrobium”*	6	6	4
*Methylosarcina*^a^	3	457	50
*Methylothermus*	3	44	30
*Methylocapsa*	3	39	7
*Methyloglobulus*	3	7	3
*Methylohalobius*	3	5	3
*Methylomarinum*	2	2	2
*Methyloparacoccus*^b^	2	422	6
*Methylovulum*	2	30	3
*Methylosoma*^c^	1	252	1
“*Candidatus* Methylomirabilis”	1	51	1
*Methylogaea*	1	24	11
*Methyloprofundus*	1	8	3
*Methylomarinovum*	1	2	1

*The number of isolates assigned to a genus is given and the total number of pmoA sequence reads in the OTUs that harbor these isolates. A strong decrease in read numbers from 12% cut-off to 4% cut-off means that isolates are different from the most frequently detected pmoA sequence types in the environment that are classified into the same OTU at genus level resolution*.

a *Includes Methylomicrobium album and Methylomicrobium agile at 12% cut-off*.

b *Includes Methylomagnum ishizawai at 12% cut-off*.

c *Includes Methylobacter tundripaludum at 12% cut-off*.

**Table 5:** **Representativeness of methanotrophic type strains at species level resolution**.

**Species**	**Cultivation-independent studies**	**Further isolates**
*Methylocystis echinoides*	694	13
*Methylocystis rosea, hirsuta*	330	53
*Methylococcus capsulatus*	273	3
*Methylocaldum tepidum*	91	1
*Methylocaldum szegediense*	74	2
*Methylosinus sporium*	49	10
*Methylosarcina lacus*	49	0
*Methylosinus trichosporium*	46	10
*Methylocaldum gracile*	45	8
*Methylobacter tundripaludum*	39	1
*Methylomicrobium buryatense, alcaliphilum, japanense*	20	1
*Methylomicrobium pelagicum*	20	0
*Methylomicrobium agile, album*	19	3
*Methylocystis parvus*	17	3
*Methylocaldum marinum*	17	0
*Methylobacter whittenburyi*	14	4
*Methylobacter marinus*	14	2
*Methylobacter luteus*	13	2
*Methylothermus thermalis, subterraneaus*	10	0
*Methylogaea oryzae*	10	0
“*Methylomonas denitrificans”*	6	10
*Methylomonas methanica*	8	0
*Methylocystis bryophila*	5	9
*Methyloparacoccus murrellii*	4	1
*Methylomicrobium kenyense*	4	0
*Methylomagnum ishizawai*	4	0
*Methylocystis heyeri*	3	1
“*Candidatus* Crenothrix”	3	0
*Methylocapsa acidiphila*	3	0
*Methylomonas lenta*	2	0
*Methyloprofundus sedimentii*	2	0
*Methyloglobulus morosus*	1	0
*Methylohalobius crimeensis*	1	0
*Methylovulum miyakonense*	1	0
*Methylobacter psychrophilus*	1	0
*Methylosarcina fibrata*	1	0
*Methylomarinum vadi*	0	2
*Methylocapsa aurea*	0	0
*Methylomarinovum caldicuralii*	0	0
*Methylomonas paludis*	0	0
*Methylosoma difficile*	0	0

*The number of reads derived from cultivation-independent studies and of further isolates that were assigned to the same OTU_4_ as the respective type strain are given*.

To evaluate how well cultivated strains cover the diversity of methanotrophic bacteria as seen in cultivation-independent studies, the distribution of their *pmoA* sequences upon OTU clustering was assessed based on the above mentioned “large *pmoA* dataset” containing 12,502 high quality sequences. The dataset includes different homologs of *pmoA* that have been detected in methanotrophs. Clustering of the sequences applying the 12 and 4% cut-off value resulted in 522 and 2287 OTUs, respectively (Table 6). In both cases, there was a rather low number of clusters with high read numbers, while one third of the OTUs_12_ or even half of the OTUs_4_ were represented by just one read (singletons). This demonstrates the existence of a very high number of taxa that are rarely detected. The percentage of OTUs that contained sequences of cultivated strains was 12 and 6%, respectively, at the different cut-off levels, which means that only a small fraction of the methanotrophic diversity is represented by cultivated strains. But remarkably, when considering the size of the OTUs_12_, it turned out that 52% of all available sequences fall into clusters that contain *pmoA* sequences of isolates. This demonstrates that half of the sequences that have been detected in cultivation-independent studies are closely related to or represented by cultivated genera. At species level, still 24% of all sequences fall into the same OTU_4_ as a cultivated strain. In conclusion, a surprisingly high number of sequence reads that are detected in environmental studies are closely affiliated to cultivated genera or species, despite the fact that the total diversity of methanotrophs that is present in nature is substantially higher than the cultured diversity.

**Table 6:** **Statistics about OTU clustering and distribution of ***pmoA*** sequences of cultivated methanotrophic strains within these clusters**.

**Cut-off:**	**12%**	**4%**
**STATISTICS OF OTU CLUSTERING**		
Number of OTUs	522	2287
Number of reads in largest cluster	2666	708
% of clusters with ≥ 100 reads	4	0.5
% of clusters with < 100 reads but ≥ 10 reads	20	10
% of singletons	36	54
**OTUs CONTAINING CULTIVATED STRAINS**		
% of OTUs with cultivated strains	11.9	6.2
% of OTUs that contain a type strain	8.2	3.0
% of OTUs that contain only cultivated strains	5.7	3.4
% of singletons represented by a cultivated strain	2.5	1.8
**REPRESENTATIVENESS OF OTUs CONTAINING CULTIVATED STRAINS**		
% of sequences in clusters with cultivated strains	52	24
% of sequences in clusters with type strains	50	17

To further evaluate the representativeness of the cultivated genera and species, the size of the OTUs harboring isolates was evaluated. The most frequently detected genera of methanotrophic bacteria in environmental studies are the alphaproteobacterial genera *Methylocystis* and *Methylosinus* and the gammaproteobacterial genera *Methylomonas, Methylobacter, Methylosarcina, Methylomicrobium, Methylococcus, Methylocaldum, Methylosoma* as well as the recently described genus *Methyloparacoccus* (Table 4). At higher taxonomic resolution, the isolated *Methyloparacoccus* species remains distinct from the related sequences that have been frequently detected in environmental samples. The same applies to *Methylosoma* and “*Candidatus* Methylomirabilis.” Further methanotrophic genera that have very rarely or not yet been detected in environmental samples via cultivation-independent methods comprise *Methylomarinovum, Methylomarinum, Methylohalobius, Methyloglobulus* and the verrucomicrobial lineages “*Methylacidiphilum*” and “*Methylacidimicrobium*” (Table 4). At lower phylogenetic resolution, the genera *Methyloglobulus* and *Methylomarinum* do serve as cultivated representatives for major uncultivated clusters (see Section Cluster 2 (CL2) or TUSC). In case of the verrucomicrobial lineages, the limited detection in environmental samples is explained by their highly divergent *pmoA* sequences, which prevents PCR amplification using the standard *pmoA* primers. At species level, the frequently detected taxa in cultivation-independent studies are *Methylocystis echinoides, Methylocystis rosea*, and *Methylocystis hirsuta*, the two *Methylosinus* species, *Methylococcus capsulatus* and most species of the genus *Methylocaldum* (Table 5). Nearly half of the validly described methanotrophic species have only rarely been detected in environmental samples based on cultivation-independent studies (<10 reads), showing that our culture collections contain many strains of which the ecological relevance in their natural ecosystems remains unknown. Remarkably, *Methylosinus* strains have very frequently been isolated, but not that frequently been detected by cultivation-independent studies. This is evident from the fact that 54% of all *Methylosinus* sequences in the database are from isolates, while most other frequently detected genera have only about 5% cultivated representatives (Table 4).

## Description of major uncultivated groups of methanotrophic bacteria and their habitat specificity

Clusters of *pmoA* sequences representing uncultivated methanotrophs have been defined in diverse studies mostly at a taxonomic resolution above genus level. They are often named according to the habitat in which they are predominantly found, the sampling site from which they were obtained, or derived from the name of the first described clones of a cluster. The assignment of sequences to a characteristic cluster is usually done in the context of phylogenetic tree reconstruction, guided by a few characteristic reference sequences that are given in the literature as representatives.

**Figure 4:**
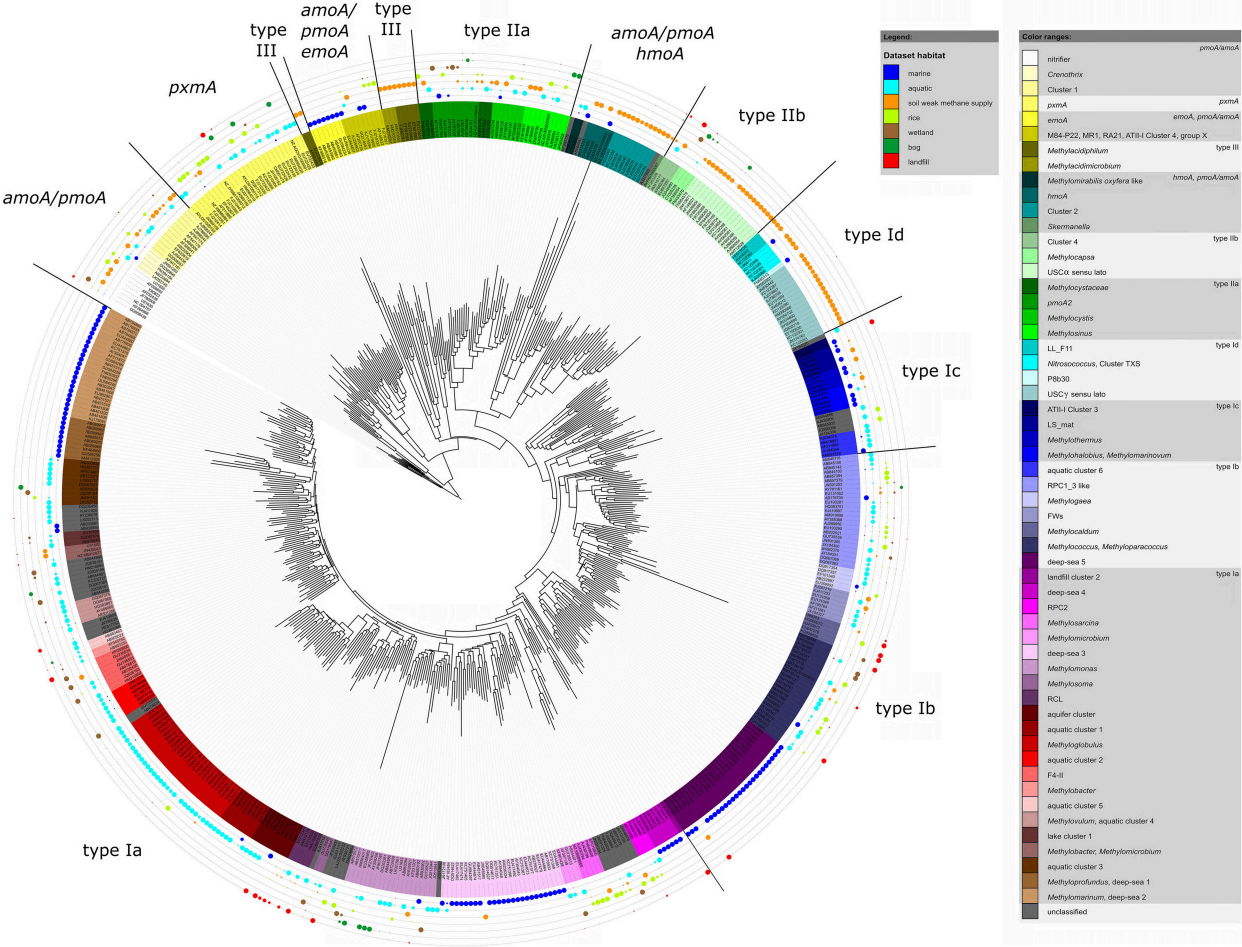
**Neighbor joining tree showing the phylogeny of representative ***pmoA*** sequences and the habitats in which they were detected**. For each OTU_12_ one representative sequence was included. The dots on the rings around the tree display detection in different habitat types. The size of the dots corresponds to the relative frequency with which sequences of an OTU_12_ were detected in the seven habitats. The diagram was set up using the iTOL online package (Letunic and Bork, 2011). The phylogenetic tree was calculated in the ARB software package based on 480 nucleotide positions and a Jukes-Cantor correction.

The same approach was used here to assign OTUs to described clusters of uncultivated methanotrophic bacteria. Neighbor joining and maximum likelihood trees were constructed using one representative sequence for each OTU_12_. These representative sequences were selected within each OTU based on the following criteria: OTUs harboring a cultivated strain were represented by the sequence of this strain. For OTUs consisting of sequences from uncultivated bacteria only, the most representative sequence from the first dataset reporting about this sequence type was taken. All representative sequences are listed along with their cluster assignment and accession number in Table S1. Uncultivated clusters were identified in the phylogenetic trees based on the position of published reference sequences. Several OTUs showed inconsistent clustering (in particular among the type I methanotrophs), they were excluded from clusters and are referred to as “incerta sedis” or by their family names and are displayed as “unknown” in Figure 4.

**Figure 5:**
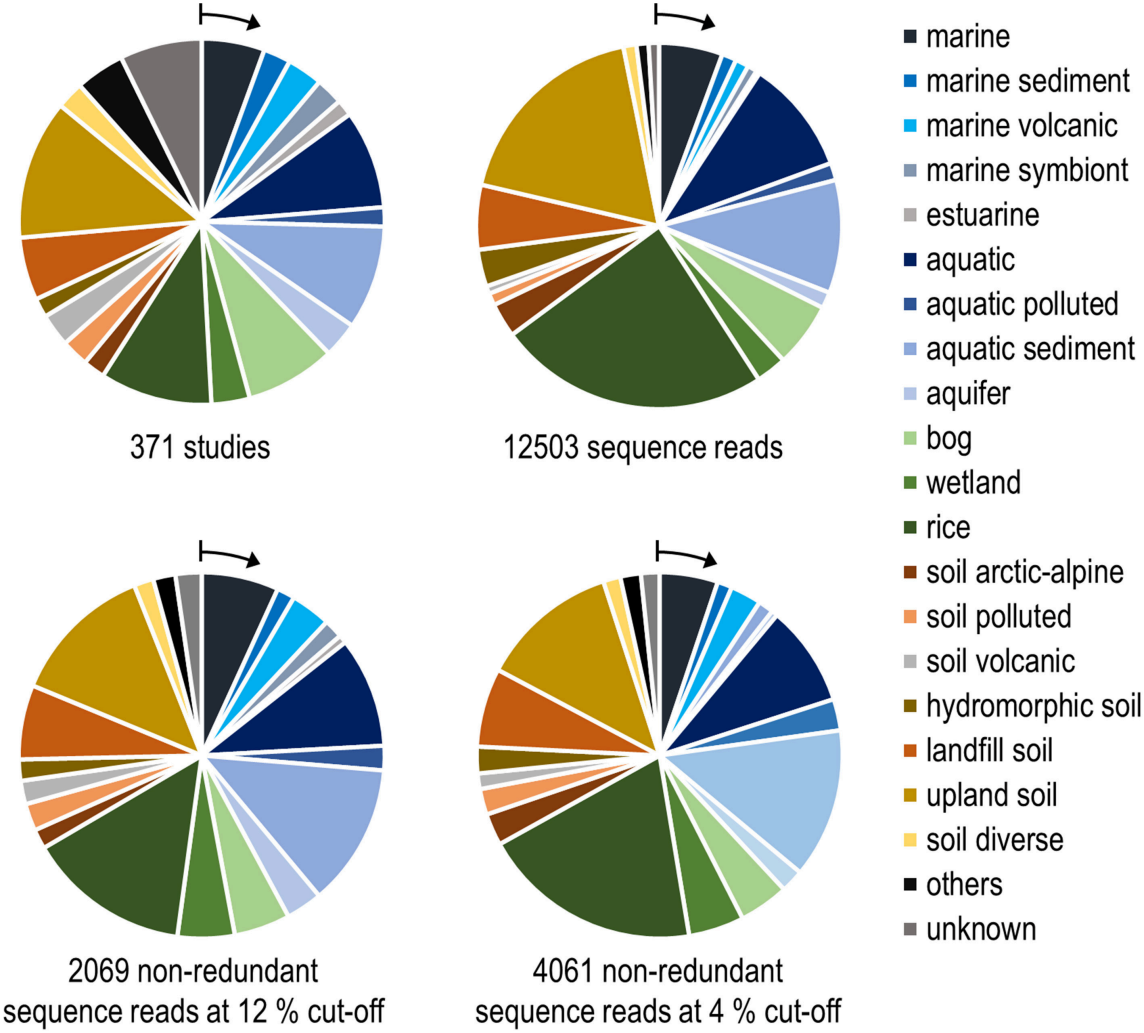
**Number of habitats that were analyzed in research studies (upper left) and grouping of ***pmoA*** sequences from the NCBI database according to the habitats in which they were detected**. The upper right diagram is based on all available high quality sequences, while the lower diagrams include only non-redundant sequence reads. Redundant reads are those that were detected in the same study and fall within the same OTU. Arrows denote the position of the group that is shown as first entry in the legend.

To integrate habitat preferences of methanotrophic lineages into the phylogenetic tree (Figure 4), information about the habitat from which sequences were obtained was collected from the literature and the NCBI database. The definition of categories was largely guided by the terminology used in the literature and the number of sequence reads obtained for each of these categories. The majority of sequences that are currently stored in the public database are from studies that analyzed methanotrophic communities in rice fields, upland soils, aquatic or marine environments (Figure 5). 2.4% of the sequences remained unclassified, either because no information about the habitat was available or they were obtained from studies analyzing rather unusual and thus little studied habitats of methanotrophs (bioreactor, manure, rumen, waste water or plants). Seven major habitat types were defined based on this information and the relative detection frequency of each OTU_12_ within these habitats calculated. The presentation of these data in combination with phylogeny allows the identification of major clusters with habitat preferences (Figure 4). Habitat specificity of individual OTUs cannot be inferred from this presentation, as a substantial number of OTUs are represented by just one sequence and thus displayed with 100% recovery from one single habitat. To evaluate this aspect, further data analysis is needed as described in Section Habitat Specificity of Methanotrophic Taxa Evaluated at Higher Taxonomic Resolution.

Remarkably, three-fourths of all OTUs_12_ represent type I methanotrophs in the phylogenetic tree (Figure 4), with nearly 50% belonging to type Ia methanotrophs. This confirms that methanotrophic diversity is highest within the *Gammaproteobacteria*. Furthermore, it is evident from Figure 4 that the methanotrophs that are found in upland soils, aquatic and marine environments form distinctive and large clusters, while the methanotrophs that are found in other habitats such as rice field soils, wetlands or landfill cover soils are found in smaller clusters that are often detected in different habitats. It is tempting to speculate that colonization of the rather young anthropogenic habitats such as rice field soils or landfill cover soils occurs via methanotrophs that evolved in the much older pristine habitats, so that evolutionary processes leading to diversification and specialization are still in a very early phase in these human made habitats. Moreover, rice field soils and wetlands may represent transitions between terrestrial and aquatic ecosystems and thus share more taxa with other habitats. The absence of specific clusters in wetlands may at least partially be the result of a rather small number of studies in which *pmoA* sequences were published for this ecosystem (Figure 5) leading to an underrepresentation of sequence reads from this habitat.

**Figure 6:**
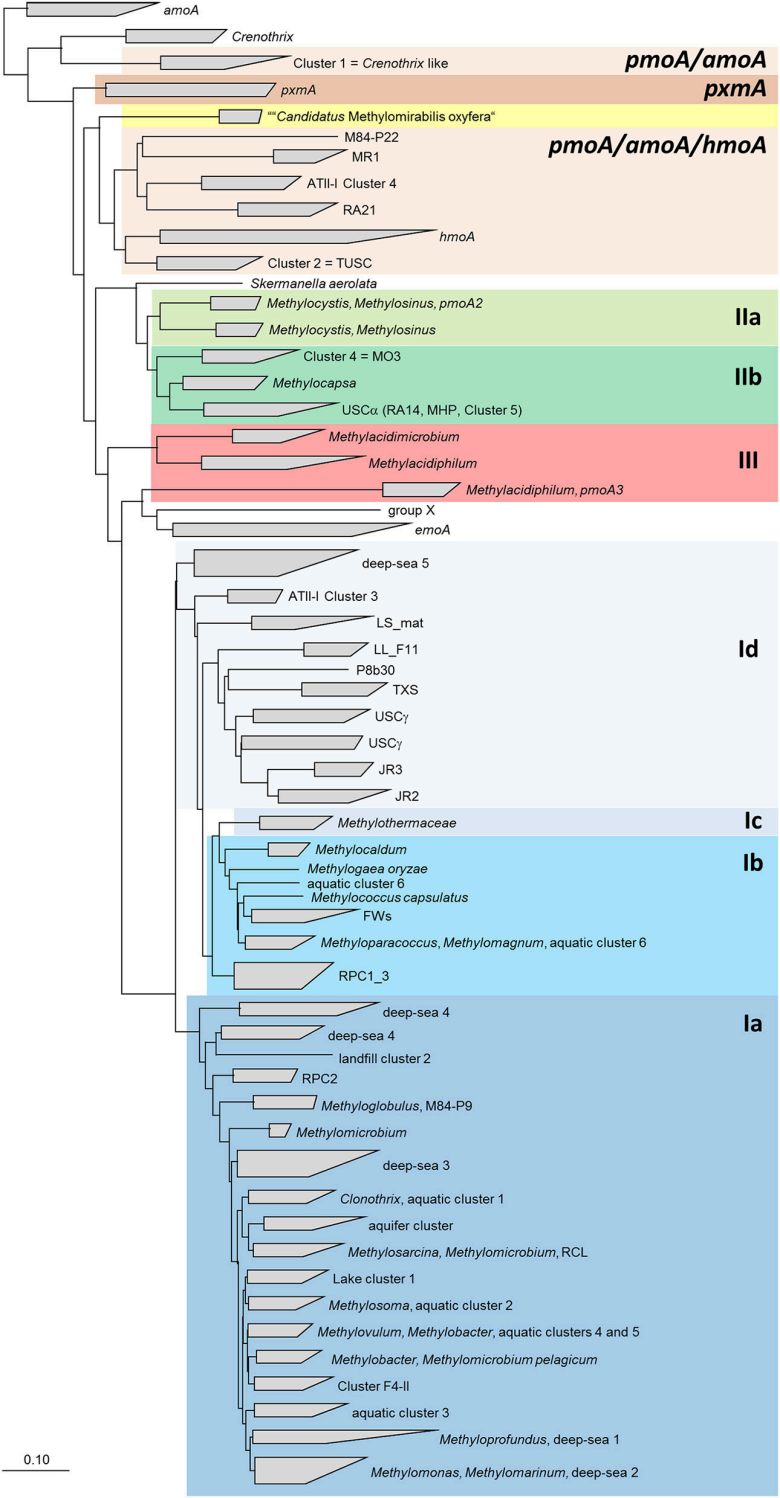
**Neighbor joining tree showing the phylogeny of uncultivated clusters in relation to methanotrophic type strains**. The tree includes *pmoA* sequences from all OTUs that were assigned to uncultivated clusters. It was calculated based on 480 nucleotide positions with Jukes Cantor correction. The scale bars display 0.10 changes per nucleotide or amino acid position.

In the following, information about the major uncultivated clusters of methanotrophs residing in different habitats is compiled. A condensed phylogenetic tree shows the phylogenetic placement of these clusters in relation to each other and to cultivated type species (Figure 6).

### Rice paddy clusters (RPC) and japanese rice clusters (JRC), including the lake washington cluster (LWs), and the organic soil cluster (OSC)

Several different rice paddy clusters and Japanese rice clusters have been defined (Lüke et al., 2010; Stralis-Pavese et al., 2011), but only some of them are regularly detected in diverse studies and implemented in phylogenetic trees. These are RPC1, 2, 3, and JRC3 as well as JRC4, which has meanwhile a cultivated representative, *Methylogaea oryzae* (Geymonat et al., 2010). JRC3, RPC1, and RPC3 are distantly related to *Methylocaldum* and *Methylococcus* and thus part of the type Ib group (Figure 6). RPC2 was reported to show variable clustering either with type Ia or Ib, depending on the algorithm used for tree reconstruction (Lüke and Frenzel, 2011). It is composed of a high number of OTUs at species level resolution, but contains only four OTUs at genus level. RPC1, RPC3, and JRC3 were combined into a larger monophyletic cluster referred to as RPC1_3 in this review, because JRC3 did not form a monophyletic cluster and could not be clearly delineated from RPC1. The RPC1_3 like cluster consists of 25 OTUs_12_, including in addition the clusters LWs and OSC. Similarly, a large cluster containing the sequences of RPC1, LWs, and OSC but without RPC3 was also formed in some other studies and referred to as freshwater lineage 1 (Lüke and Frenzel, 2011). The major habitat of the methanotrophs belonging to the RPC1_3 like cluster are rice field and aquatic ecosystems (Figure 4). RPC1 and JRC3 were initially exclusively detected in rice paddy associated habitats (Lüke et al., 2010; Lüke and Frenzel, 2011). Exceptional within the RPC1_3 like cluster is OSC, which occurs predominantly in bogs and in some upland soils (Figure 4, Tables S1–S4). Thus, the large RPC1_3 cluster is heterogeneous in terms of habitat preference, with some habitat-specific subgroups. In in-depth studies, biogeographic patterns have been shown for clusters RPC1 and JRC3 (Lüke et al., 2010). Moreover, they respond to the environmental factor rice genotype, either directly or possibly indirectly via altered physicochemical conditions in the plant rhizosphere (Lüke et al., 2011).

### Upland soil clusters (USCα and USCγ), jasper ridge clusters (JR1, JR2 and JR3), moor house peat cluster (MHP), and cluster 5

Phylogenetically, the upland soil clusters form two major groups. Sequences of USCα, JR1, and MHP (also referred to as Cluster 5) are related to *Methylocapsa* (Figure 6). USCα was initially detected by Holmes et al. (1999) and termed RA14. The name USCα was proposed for this sequence type when a second group of sequences with preferential occurrence in upland soils but related to sequences of methanotrophic *Gammaproteobacteria*, USCγ, was discovered (Knief et al., 2003). USCγ as well as JR2 and JR3 belong to the type Id group (Figure 6). These sequences are related to methanotrophic *Gammaproteobacteria* and the *amoA* sequence of *Nitrosococcus oceani*.

It was proposed to refer to the large group of USCα, JR1/Cluster 5, and MHP sequences as USCα-like sequences or USCα sensu lato, while the initially discovered RA14 clade was defined as USCα sensu stricto (Shrestha et al., 2012). Based on the sequence dataset used in this study, USCα sensu lato consists of 18 OTUs_12_ and shows an enormous diversity at lower resolution with 133 OTUs_4_ (Table 7). In particular USCα sensu stricto shows a high diversity at species level resolution. In analogy to this differentiation of USCα sensu lato, sequence clusters USCγ, JR2, and JR3 will be referred to as USCγ sensu lato in this review, while USCγ sensu stricto refers specifically to the USCγ clade. The USCγ sensu lato group is less diverse compared to USCα, consisting of 15 OTUs_12_ and 98 OTUs_4_ with USCγ sensu stricto as most diverse group, especially at species level resolution (Table 7).

**Table 7:** **Characteristics of uncultivated clusters of ***pmoA*** and ***pmoA***-like sequences^a^**.

**Cluster**	**No. of OTUs (12% cut-off)**	**No. of OTUs (4% cut-off)**	**Related to^b^**	**Major habitat**	**Representative sequences^c^**	**References**
**TYPE IA**						
Aquatic cluster 1 (incl. *Clonothrix*)	8	13	Type Ia	aquatic	AB930877, AB844864, AB845005	This study
Aquatic cluster 2 (incl. *Methylosoma*)	6	16	Type Ia	Aquatic	AB478795, KC188735, AB478808, JF811270	This study
Aquatic cluster 3	9	21	Type Ia	Aquatic	HQ383800, AB722373, JN591162	This study
Aquatic cluster 4	4	8	Type Ia	Aquatic	AY488060, HQ383801	This study
Aquatic cluster 5	2	7	Type Ia	Aquatic	AB563463, AB505022	This study
Aquifer cluster	9	13	Type Ia	Diverse	AM410175, AB930937, AM410175	Dumont et al., 2014
Deep-sea 1 (incl. OPU2, *Methyloprofundus*)	8	33	Type Ia	Marine	AB089967 (OPU1), AM089968	Hayashi et al., 2007; Lüke and Frenzel, 2011; Dumont et al., 2014
Deep-sea 2 (incl. PS-80, *Methylomarinum*)	24	68	Type Ia	Marine	AY354047, AB176934, EU444860, AB176935, AF211872 (PS-80)	Lüke and Frenzel, 2011; Dumont et al., 2014
Deep-sea 3 (incl. OPU3, EST)	27	75	Type Ia	Marine	AB276027 (OPU3), AF182484 (EST), AB276027, AB176933	Hayashi et al., 2007; Lüke et al., 2010; Lüke and Frenzel, 2011; Dumont et al., 2014
Deep-sea 4	6	9	Type Ia (Ib)	Marine	GU584278, AY354044, GU115829, FN650295	Lüke and Frenzel, 2011; Dumont et al., 2014
F4-II	8	18	Type Ia	Diverse	AB478819, GU735534, HM216892	This study, referring to Chauhan et al., 2012
Lake cluster 1	3	17	Type Ia	Diverse	AB478843, DQ067079	Dumont et al., 2014
Landfill cluster 2	1	2	Type Ia	Landfill soil	EU275101	Dumont et al., 2014
RCL	5	47	Type Ia	Diverse	EF212356, EF212340, HM216885	Chen et al., 2007
RPC2	4	51	Type Ia (Ib)	Rice, aquatic	FN600101, EU358980	Lüke et al., 2010; Dumont et al., 2014
**TYPE IB**						
Aquatic cluster 6 (incl. *Methyloparacoccus* and *Methylomagnum*)	6	15	Type Ib	Aquatic	AF211880, JX184344, AB478851	This study
Deep-sea 5 (incl. OPU1)	28	54	Type Ib/c/d	Marine	AB276025 (OPU1), AY354045, AB176940, FN650305, EU417532	Hayashi et al., 2007; Lüke and Frenzel, 2011; Dumont et al., 2014
FWs	4	50	Type Ib	Aquatic	AF211881, AF150764, EU131048	Lüke and Frenzel, 2011; Dumont et al., 2014
RPC1_3 like (incl. RPC1, RPC3, LWs, JRC3, OSC)	25	178	Type Ib	Diverse	AJ299956 (RPC1), EU193281 (RPC3), DQ067069 (LWs), AY355388 (JRC3), AY781161 (OSC), AB845118	Lüke et al., 2010; Lüke and Frenzel, 2011; Dumont et al., 2014
**TYPE IC AND ID**						
ATII-I Cluster 3	2	2	Type Ic/d	Marine	KJ175590, EU275114	This study, referring to Abdallah et al., 2014
LL_F11	3	5	Type Id	Upland soil	HE613038, KC122282	This study
LS_mat	5	12	type Ic/d	Marine, upland soil	JF780903, FR670562, JF780909	This study, referring to Crépeau et al., 2011
TXS	4	21	Type Id	Upland soil	KC122309, KJ026966, KC122329	This study, referring to Serrano-Silva et al., 2014
USCγ, sensu stricto	7	63	Type Id	Upland soil	AJ579667, AY662351, KC122280	Knief et al., 2003
USCγ, JR2	6	17	Type Id	Upland soil	AY654695, KC122283, KC122301	Horz et al., 2005
USCγ, JR3	3	18	Type Id	Upland soil	AY654702, KM390988	Horz et al., 2005
**TYPE IIB**						
Cluster 4 = MO3	4	10	Type IIb	Diverse	AF283229, AM410177	Henckel et al., 2000b; Knief et al., 2006
USCα, sensu stricto = RA14	4	72	Type IIb	Upland soil	AF148521, EF015805	Holmes et al., 1999; Knief et al., 2003
USCα, MHP	4	25	Type IIb	Upland soil	EF644609, AJ868263	Chen et al., 2008
USCα, JR1 = Cluster 5	10	36	Type IIb	Upland soil	AJ868264, AY662381	Horz et al., 2005; Knief et al., 2005
***pmoA/amoA***						
ATII-I Cluster 4	2	2	*pmoA/amoA*	Marine	KJ175600, KJ175594	This study, referring to Abdallah et al., 2014
Cluster 1 = *Crenothrix* related	12	47	*pmoA/amoA*	Upland soil	AF358041, AJ868244, AF547181, AF547179	Kolb et al., 2005; Ricke et al., 2005; Lüke and Frenzel, 2011
Cluster 2 = TUSC	10	21	*pmoA/amoA*	Upland soil	AJ579663, AJ868246, EU723743, KC122308	Knief et al., 2005; Ricke et al., 2005; Lüke et al., 2010
M84-P22	1	2	*pmoA/amoA*	Diverse	AJ299963	Horz et al., 2001
MR1	2	3	*pmoA/amoA*	Upland soil	AF200729, GQ219583	Henckel et al., 2000a
RA21	3	29	*pmoA/amoA*	Rice	AF148522, FJ210291, FJ210332	Holmes et al., 1999

a *The number of different OTUs per cluster given in this table should be considered as estimate reflecting the diversity within each cluster. The assignment of sequences to clusters was evaluated by comparison of trees calculated based on different algorithms and sequence input datasets, but it cannot be excluded that further variation in treeing algorithms and input data may lead to slightly different results in terms of clustering. This applies in particular to type Ia clusters*.

b *Assignment to type Ia, Ib, Ic, or Id is in some cases uncertain as it varies depending on the tree reconstruction algorithm and sequence dataset*.

c *Reference sequences were selected from those OTUs that were most frequently detected in different studies, reflected the diversity of the cluster as good as possible and showed robust results during phylogenetic tree reconstruction. A complete list of sequences assigned to each cluster based on the results in Figure 4 is given in Table S1*.

All upland soil cluster sequences occur in soils, predominantly in upland soils. USCα sensu lato has been identified as dominant *pmoA* type in different forest soils (Kolb et al., 2005; Degelmann et al., 2010; Dörr et al., 2010). Some USCα sequence types have additionally been detected in hydromorphic soils (Figure 4, Tables S1–S4) (Knief et al., 2006; Shrestha et al., 2012). USCγ sensu lato occurs in pH neutral and alkaline soils and has been reported to dominate in soils collected from an alpine meadow, an arid desert ecosystem and a former lake (Angel and Conrad, 2009; Zheng et al., 2012; Serrano-Silva et al., 2014). Moreover, USCγ OTUs have been detected sporadically in landfill cover soils (Kumaresan et al., 2009; Henneberger et al., 2012). The occurrence of the two upland soil clusters is clearly pH dependent. USCα sensu lato occurs in acidic to pH neutral soils, while USCγ is only detected in pH neutral and alkaline soils (Knief et al., 2003; Kolb, 2009).

The occurrence of the USC methanotrophs is in most soils reduced the more intensively a soil is agriculturally managed. The clusters are consistently found in forest soils, often as most abundant group, they are quite frequently detected in grassland soils, but rarely detected in intensively managed agricultural soils (Knief et al., 2006; Dunfield, 2007). It has been reported that populations decrease and become inactive when forest soils are converted into agricultural soils, or grasslands are subjected to grazing (Knief et al., 2005; Abell et al., 2009; Dörr et al., 2010; Lima et al., 2014). They recover in afforested or reforested sites and grassland soil in which nitrogen fertilization is reduced (Nazaries et al., 2011; Shrestha et al., 2012). The data of Degelmann et al. (2010) suggest that habitat specificity may exist within USCα sensu lato, as some OTUs occurred in deciduous but not in spruce forest soils.

The USC methanotrophs are assumed to be involved in the oxidation of atmospheric methane (Dunfield, 2007; Kolb, 2009), but this might be different for one specific OTU within USCα sensu lato. OTU 75 (USCα 5, MHP) has more frequently been detected in soils with higher methane supply, i.e., peatlands and wetland, than in typical upland soils (Tables S1–S4) (Chen et al., 2008; Liebner and Svenning, 2013; Yun et al., 2015). Initially it was assumed that the USC methanotrophs may obtain enough energy from atmospheric methane oxidation for cell maintenance and growth (Knief and Dunfield, 2005; Kolb et al., 2005), but later calculations based on methane uptake rates and estimated cell numbers in forest soils indicated that an additional energy source is needed for survival (Degelmann et al., 2010). Indeed, it could be proven that ^13^C-labeled acetate is incorporated into the biomass of USCα methanotrophs, suggesting that these are facultative methanotrophs (Pratscher et al., 2011).

### Cluster 4 (CL4) or MO3

Besides USCα sensu lato, only one further cluster of sequences representing an uncultivated group of methanotrophs is known among the type II group. This is Cluster 4, also known as MO3. It consists of only four OTUs_12_, is related to *Methylocapsa* and was initially detected in rice field soil (Henckel et al., 2000b). Upon repeated detection it was defined as cluster 4 (Knief et al., 2006). The cluster has been detected quite frequently in diverse soil habitats including landfill cover, hydromorphic, upland and wetland soils (Figure 4, Tables S1–S4). Its growth was stimulated when rice field soil was incubated under high methane and oxygen concentrations (Henckel et al., 2000b).

### Cluster 1 (CL1) or *Crenothrix* related cluster

A sequence cluster related to *pmoA* of *Crenothrix, amoA* of nitrifying bacteria and hydrocarbon monooxygenases (*hmoA, emoA*) was described as cluster 1 (Kolb et al., 2005; Ricke et al., 2005; Knief et al., 2006; Lüke and Frenzel, 2011). It was later also referred to as *Crenothrix* related cluster (Lüke and Frenzel, 2011). Cluster 1 contains some sequences from methanotrophic isolates that were obtained from Canadian Arctic soils (Pacheco-Oliver et al., 2002). Based on their 16S rRNA gene sequences, these isolates are related to *Methylocystis* and *Methylosinus*. Unfortunately, the isolates have been lost and similar isolates could so far not be obtained again, so that the identity and characteristics of the bacteria harboring this *pmoA* sequence type remain unclear. It has been speculated that cluster 1 organisms are responsible for atmospheric methane uptake, as they were detected as dominant *pmoA* sequence type in some upland soils, in particular in pH neutral soils (Kolb et al., 2005; Ricke et al., 2005; Kolb, 2009). Experimental proof for this hypothesis is still missing. Further sequences assigned to Cluster 1 were detected in aquatic sediments and aquifers (Figure 4, Tables S1, S3). This corresponds well to the habitat of the related *Crenothrix* organisms, which were enriched from backwash water of sand filters fed with ground water (Stoecker et al., 2006). Thus, at least some Cluster 1 organisms may be similar to *Crenothrix* and the whole cluster appears to harbor methanotrophs adapted to different habitats.

### Cluster 2 (CL2) or TUSC

Another sequence cluster with *pmoA/amoA* like sequences was referred to as cluster 2 upon its recurring detection (Knief et al., 2003, 2005; Ricke et al., 2005). In later studies it was named tropical upland soil cluster (TUSC) (Lüke et al., 2010), though its occurrence is not restricted to tropical soils. Instead, it has been detected in diverse upland soils and some hydromorphic soils. It shows similarities in dispersal to USCγ, as it is largely absent in wetlands and acidic soils (Kolb, 2009; Martineau et al., 2014). Moreover, it shows reduced occurrence in intensively managed agricultural soils (Lima et al., 2014) with the exception that it has been found in some agricultural fields that are subjected to organic farming and/or that are characterized by higher carbon content (upon biochar or organic residue application; Dörr et al., 2010; Lima et al., 2014; Ho et al., 2015).

It has been speculated that the organisms harboring genes of this sequence cluster are involved in atmospheric methane oxidation, but this is solely based on the specific detection of this sequence type in upland soils. Further proof for this hypothesis is missing. It can currently not even be excluded that the genes of this sequence cluster encode a non-methane hydrocarbon monooxygenase, which is suggested by the fact that the sequences are related to those of hydrocarbon monooxygenases (Figure 6). The only evidence that supports the assumption that cluster 2 sequences may represent methanotrophic *Gammaproteobacteria* comes from a study of Kalyuzhnaya et al. (2006), who enriched methanotrophic bacteria from lake Washington sediment by cell sorting using 16S rRNA targeted fluorescent probes. Twenty percent of a *pmoA* clone library, constructed from a cell suspension enriched with a probe for type I methanotrophs, represented cluster 2 *pmoA* sequences. Unusual in this context remains the unique detection of this sequence type in a lake sediment.

### Deep-sea clusters 1 to 5 including OPU1, OPU3, and PS-80

Sequences retrieved from marine environments can be grouped into five major clusters, referred to as deep-sea clusters 1 to 5 (Lüke and Frenzel, 2011). Deep-sea clusters 1, 2, and 3 belong to the type Ia methanotrophs (Figure 6). Deep-sea cluster 4 is distantly related to known type Ia and Ib methanotrophs. Depending on the subset of sequences and the method used for tree reconstruction this cluster falls within either type Ia or type Ib methanotrophs (Lüke and Frenzel, 2011). Deep-sea cluster 5 is a deeply branching lineage related to type Ib and Ic methanotrophs. The clustering is variable in different phylogenetic trees, so that an unambiguous assignment to one or the other type is difficult. In some studies, this cluster was even assigned to type Id (referred to as type Ic in those studies; Lüke and Frenzel, 2011; Henneberger et al., 2012).

Deep-sea clusters 1 and 2 have meanwhile cultivated representatives. Cluster 1 includes the cultivated genus *Methyloprofundus* and cluster 2 the genus *Methylomarinum*. These genera represent one single OTU within the respective clusters, while the clusters contain in total eight and 27 OTUs_12_. Thus, it appears likely that they consist of more than one genus. Hence, the well-established names deep-sea cluster 1 and 3 are kept for these larger clusters of sequences in this review. Deep-sea cluster 2 includes the uncultivated PS-80 cluster, which is displayed as distinct cluster in some phylogenetic trees or given as alternative name for deep-sea cluster 2 (Deng et al., 2013; Dumont et al., 2014; Li et al., 2014). Likewise, deep-sea cluster 3 includes the sub-clusters OPU3 and EST, which are repeatedly mentioned in the literature and sometimes given as synonym for deep-sea cluster 3 (Lüke et al., 2010; Tavormina et al., 2010, 2013; Crespo-Medina et al., 2014; Li et al., 2014). The same applies to deep-sea cluster 5, which includes or corresponds to OPU1.

Deep-sea clusters 1 and 4 are rather small with only 6 and 8 OTUs_12_ and have less frequently been detected compared to the other three clusters, which contain between 20 and 30 OTUs_12_ (Table 7). Most deep-sea clusters consist exclusively of sequences from marine habitats, the only exceptions are found in deep-sea clusters 3 and 5 (Figure 4). They contain one single OTU_12_, which was retrieved from a terrestrial habitat, i.e., a mud volcano and a landfill cover soil (Henneberger et al., 2012). Furthermore, OTU 271 in cluster 3 contains some sequences from an aquatic habitat. These were detected in an estuarine sediment, which harbored otherwise sequences that are typical for aquatic habitats (McDonald et al., 2005).

Possible habitat preferences of the different deep-sea clusters remain currently largely unknown. In most studies, sequences of two or more deep-sea clusters have been detected in the same sample (Nercessian et al., 2005; Yan et al., 2006; Redmond et al., 2010; Ruff et al., 2013). Nevertheless, methanotrophic communities can differ substantially between sites (Ruff et al., 2013). Clear differences were also seen between sediment and water column within the same site (Tavormina et al., 2008), but overall, all five clusters have been detected in samples from the water column or the sediment with roughly equal frequency. Evidence for habitat specificity is only seen within deep-sea cluster 1, which harbors the majority of sequences that were found in association with marine animals (Zbinden et al., 2008; Wendeberg et al., 2012; Raggi et al., 2013). These methanotrophs live as endosymbionts in mussels, tube worms or shrimps and contribute to the food web of deep-water ecosystems (Petersen and Dubilier, 2009). Deep-sea cluster 2 and 4 sequences have also been detected as endosymbionts or epibionts of marine animals, but less consistently (Zbinden et al., 2008; Rodrigues et al., 2011; Watsuji et al., 2014).

### Lake cluster 1, aquifer cluster, and aquatic clusters 1 to 6

Sequence types that have predominantly been detected in aquatic habitats are grouped into lake cluster 1 and 2 and the aquifer cluster (Dumont et al., 2014). Lake cluster 1 is a small group of sequences (3 OTUs_12_) belonging to the type Ia methanotrophs (Figure 6). Most lake cluster 1 sequences were detected in aquatic ecosystems, while few were found in a wetland. Lake cluster 2 sequences represent also type Ia methanotrophs and were grouped by the Mothur classification tool into one single large OTU together with *Methyloparacoccus*. Thus, it is referred to as *Methyloparacoccus* here instead of lake cluster 2. This OTU was not only detected in aquatic ecosystems, but also in rice ecosystems and sporadically in other habitats (Figure 4).

The aquifer cluster consists of nine OTUs_12_ and just a few more OTUs at species level resolution. It is also representing type Ia methanotrophs. The name refers to the initial detection in a petroleum-contaminated aquifer (Urmann et al., 2008), but sequences of this cluster occur in different habitats. Half of the OTUs_12_ are common in aquatic ecosystems, while others were detected in landfill cover soils (Figure 4, Tables S1, S2). This applies even to the OTU harboring the aquifer sequences; it was also detected in landfill cover soils.

The evaluation of the relationship between phylogeny and habitat revealed the existence of possible further aquatic clusters that were defined in this work (Figure 4). The aquatic clusters 1 to 5 are related to type Ia methanotrophs, while aquatic cluster 6 is a member of the type Ib methanotrophs. Aquatic cluster 1 is related to *Clonothrix*, aquatic cluster 2 to *Methylosoma*, and cluster 4 often includes *Methylovulum* in phylogenetic trees. All clusters are rather small, consisting of two to nine OTUs_12_ (Table 7). They contain dominantly sequences from aquatic ecosystems plus some sequences from other habitats, often from marine ecosystems (Figure 4). Most aquatic clusters and the lake cluster 1 OTUs were detected in samples from the water column as well as the sediment. Only aquatic cluster 4 shows a much higher detection frequency in studies of sediment samples, while cluster 2 shows a higher detection frequency in samples from the water column (Tables S1–S4). Similarly, the aquifer cluster has not yet been detected in aquatic sediment samples.

### Further clusters of uncultivated gammaproteobacterial methanotrophs

Two further clusters of uncultivated methanotrophs are related to type Ia methanotrophs, represented by cluster RCL and F4-II. Cluster RCL was named after the first clones, obtained during a study analyzing active methanotrophs in landfill cover soil (Chen et al., 2007). It consists of only five OTUs_12_, but a much higher number of 47 OTUs_4_ at higher taxonomic resolution (Table 7). It has been detected in different ecosystems, in particular in aquatic sediments and landfill cover soils (Tables S1–S4, Figure 4). Cluster F4-II was defined in this work, referring to the first study in which this sequence type was discovered (Chauhan et al., 2012). It consists of eight OTUs_12_ and contains sequences from diverse habitats, especially aquatic and wetland ecosystems.

Cluster FWs is present within the type Ib methanotrophs and was defined recently (Dumont et al., 2014). It has a relatively high diversity at species level and has most frequently been detected in aquatic environments.

Two rather small clusters of uncultivated methanotrophs, clusters LS-mat and ATII-I cluster 3 can be assigned to the type Ic or Id methanotrophs, depending on the treeing approach (Figures 4, 6). These clusters were named in this work in accordance with the sample and cluster names given in the studies in which they were first described (Crépeau et al., 2011; Abdallah et al., 2014). They are closely related to each other and were detected in different marine studies and with lower frequency in some terrestrial habitats.

Besides USCγ sensu lato one further cluster of uncultivated sequences is present within the group of type Id methanotrophs. The TXS cluster consists of four OTUs_12_ and has been exclusively detected in upland soils so far, likewise as the other uncultivated type Id clusters (Serrano-Silva et al., 2014). Whether the organisms of this cluster are also involved in atmospheric methane oxidation is unknown.

### Further *pmoA/amoA* like clusters: MR1, RA21, and others

Several further sequence types form small clusters that are distantly related to the well-known *pmoA* and *amoA* sequences as well as to those of *pxmA* and non-methane hydrocarbon monooxygenase genes. Cluster MR1 is represented by two OTUs_12_ in this study and has only been detected in some upland soils (Table 7). In contrast, RA21, which has been more frequently retrieved and consists of three OTUs_12_, is predominantly found in rice field soils. Some further clusters have been defined in this region of the phylogenetic tree, such as the two marine clusters referred to as group X (Tavormina et al., 2010) and ATII-I Cluster 4 (Abdallah et al., 2014), or cluster M84-P22 (Horz et al., 2001). These clusters have until now only been detected very rarely, so that it is too early to draw further conclusions about possible habitat preferences.

## Habitat specificity of methanotrophic taxa evaluated at higher taxonomic resolution

To evaluate habitat specificity for cultivated and uncultivated taxa of methanotrophic bacteria in more detail and at higher taxonomic resolution, 19 different habitat types were defined, which contained at least 30 sequence reads. The assignment of sequences to one of these more specific habitat types was in most cases unambiguous, but for the soil categories an overlap between habitats may exist. This applies for instance to soils collected in arctic-alpine environments, which include samples from glacier forefields as well as alpine meadows and grasslands. Some of these soils may also represent the category “upland soil” or “hydromorphic soil.” Likewise, a polluted soil may at the same time be an “upland soil.” Soils in the category “polluted soils” were collected from areas with hydrocarbon pollution, near coal mines or above oil and gas reservoirs. Four percent of the soil derived sequence reads could not be assigned to a specific soil habitat since no further information about the type of soil habitat was available. These sequences are presented as “soil diverse” in Figure 5, but were excluded from subsequent analyses as they formed a very heterogeneous group. Certain overlap may also exist between wetlands and bog ecosystems, as it cannot be fully excluded that the term wetland was in some cases used by authors for the description of samples from bog ecosystems.

**Figure 7:**
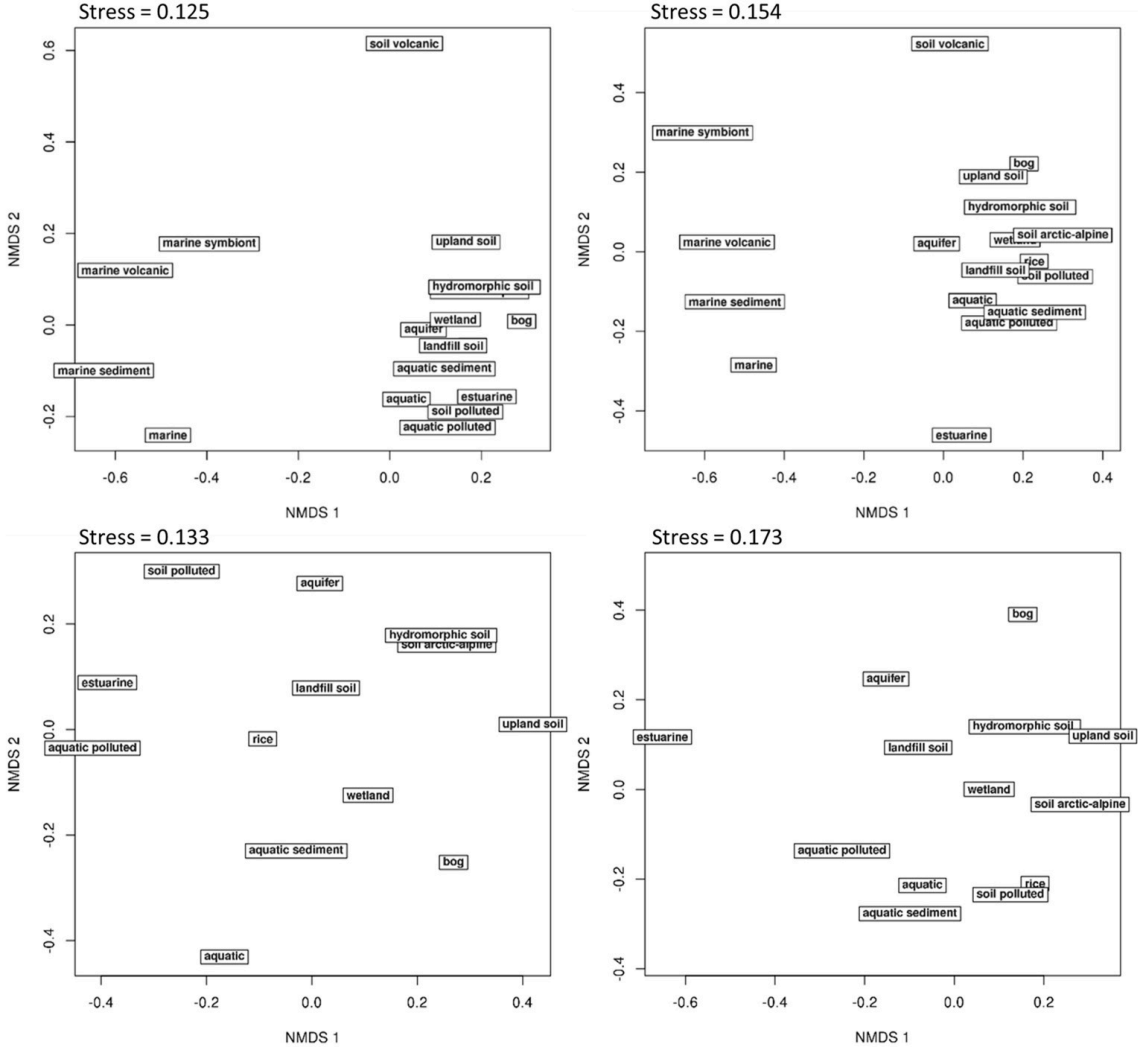
**Habitat specificity of methanotrophic bacteria evaluated in non-metric multidimensional scaling (NMDS) plots**. Non-redundant sequence reads were used to calculate the relative detection frequency of all OTUs in a habitat. The upper plots show differences between all 18 different habitats, while the lower plots focus on the 13 most similar habitats. OTU clustering was done using 12% (left panels) and 4% dissimilarity cut-off (right panels). The NMDS plots were set up based on Bray-Curtis dissimilarities calculated from Hellinger transformed data using the online tool GUSTA ME (Buttigieg and Ramette, 2014).

Due to the fact that methanotrophic communities were analyzed at very great depth in some studies, numerous redundant reads are present in the database and a high number of sequence reads assigned to certain OTUs may be the result of just a few studies rather than frequent detection in diverse studies. To correct for this possible artifact, replicate sequence reads, i.e., those that represent the same study and the same OTU, were excluded during the further analysis. This resulted in 2079 non-redundant reads at OTU_12_ cut-off and 4061 reads at OTU_4_ cut-off level. In particular at 12% cut-off, this caused a more even distribution of sequence reads across the different habitat types (Figure 5). The recovery of specific sequence types in different habitats was thus evaluated based on their presence or absence in individual studies, while the information from approximately 370 studies was used to estimate the detection frequency of each OTU quantitatively. Non-metric multidimensional scaling plots were calculated to visualize (dis-)similarities between habitats (Figure 7). The major pattern was largely similar, regardless of the applied OTU resolution, demonstrating that major differences between samples are indeed already manifest at genus level. Methanotrophic communities in marine habitats are most distinct from those of all other habitats, as evident from their clear separation along the first axis of the plot. This was also seen when applying other multivariate approaches (principal component analysis, hierarchical cluster analysis) and is in agreement with the existence of the very specific marine clades deep-sea clusters 1 to 5. The second axis separates volcanic soils from all other samples, which can be explained by the unique presence of *Verrucomicrobia* in several of these soils (Sharp et al., 2014). The high dissimilarities of methanotrophic communities in marine ecosystems and volcanic soil samples compared to all other ecosystems were verified by an analysis of similarity (ANOSIM), which revealed very high values of *R* = 0.985 (*P* = 0.001) at OTU_12_ level and of *R* = 0.926 (*P* = 0.001) at OTU_4_ level. To better evaluate dissimilarities between the remaining aquatic and terrestrial ecosystems, the marine and volcanic soil sample data were excluded from NMDS plots (Figure 7). These reduced datasets reveal that aquatic habitats including the estuarine habitat are again distinct from the other habitats, supported by ANOSIM values of *R* = 0.444 (*P* = 0.006) for OTU_12_ and *R* = 0.460 (*P* = 0.004) for OTU_4_. This agrees with the existence of different aquatic clades (Figure 4). Methanotrophic communities in aquifers appear to be somewhat different from those in aquatic habitats (Figure 7). The terrestrial samples did not show highly consistent patterns in the NMDS plots (or in other multivariate approaches), besides the observation that those soils that are exposed to low methane concentrations, i.e., upland soils, arctic-alpine soils, and hydromorphic soils, are often located close to each other. This is in agreement with the unique occurrence of the upland soil clusters and some other clades in these soils (Figure 4). The limited resolution of differences between the different soil sample types may be related to the fact that these categories may partially overlap, as explained above.

### Habitat-specific OTUs

**Figure 8:**
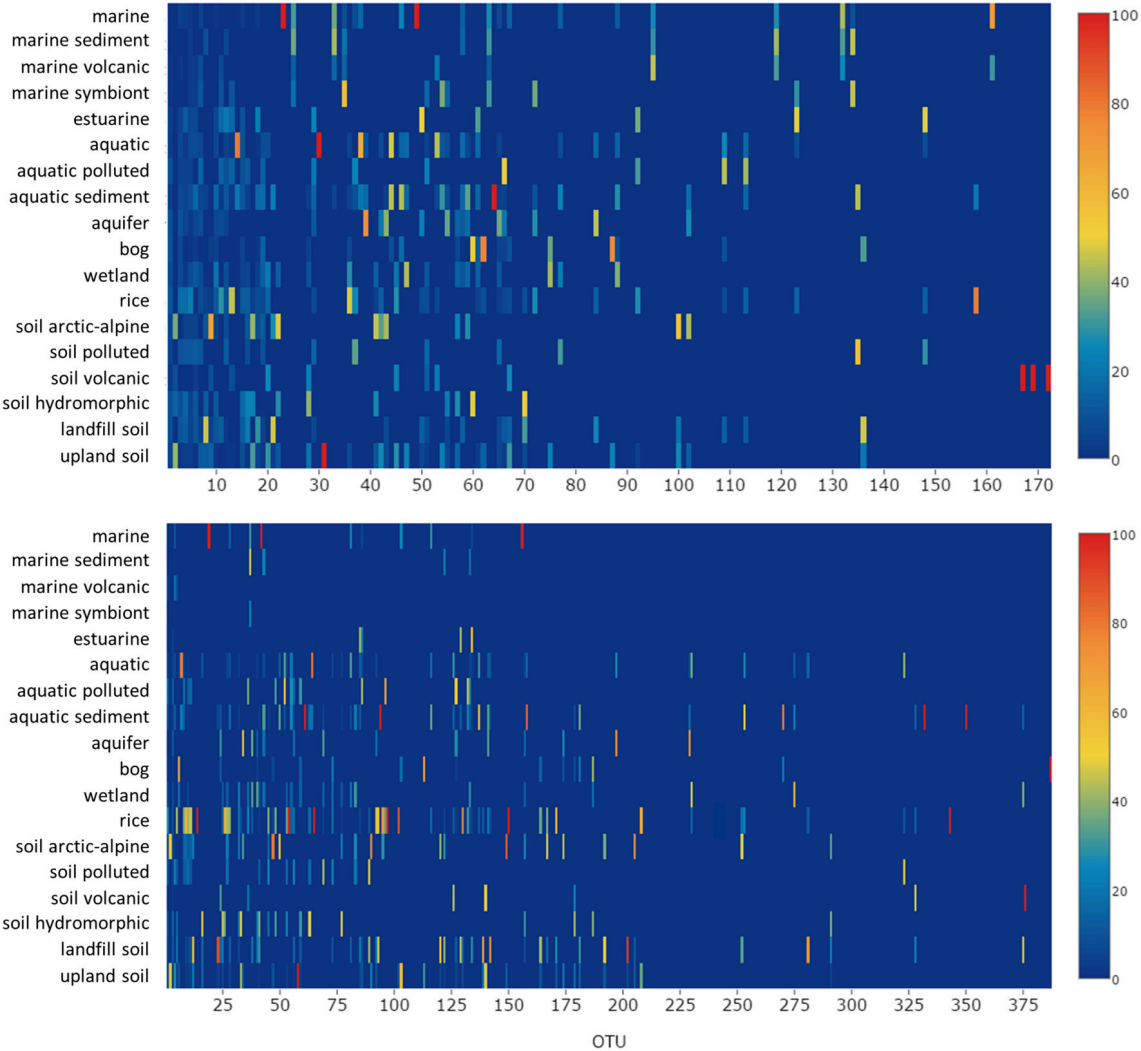
**Relative detection frequency of OTUs across habitats**. Non-redundant reads were normalized by the number of studies available for each habitat and the relative frequency with which each OTU was detected across the different habitats was calculated. The upper panel shows the results at genus level resolution (OTU_12_), the lower panel at species level resolution (OTU_4_). OTUs displayed in red are highly specific for a certain type of habitat. OTUs that were detected in less then five studies were set to zero. The identity of the most habitat-specific and most common OTUs is given in Tables 8, 9. A list including detailed information about all OTUs is provided as Supplementary Material.

To identify common and habitat specific groups at OTU_12_ and OTU_4_ level, the relative detection frequency of OTUs across habitats was determined based on non-redundant read counts. OTUs that were detected in at least five studies were included in this evaluation. Otherwise, OTUs may appear erroneously as habitat-specific based on the fact that they have been detected in a limited number of studies. The detection frequency of OTUs across habitats is displayed as heat map and reveals that a rather low number of OTUs is highly habitat specific (Figure 8). The identified habitat specific and common OTUs are listed in Tables 8, 9. The number of specific OTUs increases at species level resolution. This is to some extent the result of splitting a habitat specific genus into several habitat specific species. Furthermore, it is based on the fact that some habitat specific species exist within genera that show a broad distribution, as observed for some *Methylocystis* species. The genus is commonly found in diverse environments, but some *Methylocystis* species show habitat specificity and appear to be characteristic for aquatic environments or landfill cover soils (Tables 8, 9). Likewise, the genus *Methylocaldum* has been detected in diverse habitats, while the species *Methylocaldum gracile* was found with very high frequency in landfill cover soils.

**Table 8:** **Broadly distributed and habitat-specific OTUs_12_**.

**OTU**	**Name of cluster**	**Marine**	**Aquatic**	**Upland soils**	**Rice**	**Wetland**	**Bog**	**Soil, landfill**	**Number of non-redundant sequences**
**COMMON, CULTIVATED**									
1	*Methylocystis*	2	23	23	20	5	16	9	175
3	*Methylomonas*	6	29	11	31	4	8	11	81
4	*Methylosarcina, Methylomicrobium album/agile*	4	25	20	36	2	0	14	62
5	*Methyloparacoccus*	6	30	14	39	5	2	5	68
6	*Methylobacter psychrophilus*	4	51	16	2	8	6	12	51
7	*Methylococcus*	14	29	33	14	5	0	5	28
8	*Methylocaldum*	4	8	35	8	0	0	46	33
10	*Methylosoma, Methylobacter tundripaludum*	2	40	10	20	12	7	10	62
15	*Methylosinus*	2	36	21	19	2	7	12	49
18	*Methylobacter luteus/whittenburyi/marinus*	3	34	14	14	3	3	29	40
65	*Methylovulum*	8	38	23	8	8	8	8	14
66	*Methylocystis, pmoA2*	0	47	11	16	0	11	16	19
67	*Methylomicrobium pelagicum*	10	0	60	10	0	10	10	11
**COMMON, UNCULTIVATED**									
12	FWs 1a	4	50	14	21	7	0	4	29
16	RCL a	0	14	24	29	5	0	29	22
19	RPC1_3 like 1, LWs	0	44	12	12	8	20	4	25
21	RA21	0	10	60	20	10	0	0	10
36	*Methylocystaceae* 11	0	12	12	59	12	6	0	17
51	RPC1_3 like 8, JRC 3	10	20	30	20	10	0	10	10
61	RPC1_3 like 2, LWs	0	58	8	8	8	17	0	12
**SPECIFIC, CULTIVATED**									
134	*Methyloprofundus*	100	0	0	0	0	0	0	5
167	*“Methylacidiphilum fumarolicum/kamchatkense,“ pmoA1*	0	0	100	0	0	0	0	5
169	*“Methylacidiphilum fumarolicum/kamchatkense,“ pmoA2*	0	0	100	0	0	0	0	5
172	*“Methylacidiphilum fumarolicum/kamchatkense,“ pmoA3*	0	0	100	0	0	0	0	5
158	*Methylosinus trichosporium, pmoA2*	0	20	0	80	0	0	0	5
60	*Methylocapsa acidiphila*	0	0	17	0	0	83	0	6
**SPECIFIC, UNCULTIVATED**									
23	Deep-sea cluster 3p, OPU3	100	0	0	0	0	0	0	5
25	Deep-sea cluster 2r	100	0	0	0	0	0	0	8
35	Deep-sea cluster 1d	100	0	0	0	0	0	0	12
49	Deep-sea cluster 5w, OPU1	100	0	0	0	0	0	0	7
63	Deep-sea cluster 2t	100	0	0	0	0	0	0	9
95	Deep-sea cluster 5h	100	0	0	0	0	0	0	5
119	Deep-sea cluster 2q	100	0	0	0	0	0	0	7
132	Deep-sea cluster 2g	100	0	0	0	0	0	0	5
161	Deep-sea cluster 5d	100	0	0	0	0	0	0	5
33	Deep-sea cluster 3q	88	13	0	0	0	0	0	8
14	Lake cluster 1a	0	100	0	0	0	0	0	9
30	Aquatic cluster 5a	0	100	0	0	0	0	0	5
39	*Methyloglobulus* like 13, LP20	0	100	0	0	0	0	0	6
64	Aquatic cluster 4a	0	100	0	0	0	0	0	8
44	*Methylococcaceae* 12d	0	92	0	0	0	8	0	13
38	Aquatic cluster 2b	11	89	0	0	0	0	0	9
2	USCα 4, RA14	0	0	100	0	0	0	0	26
31	JR3a	0	0	100	0	0	0	0	7
17	USCα 16, JR1, Cluster 5	0	0	93	7	0	0	0	14
22	USCα 8, MHP	0	0	86	0	14	0	0	7
9	USCγ 1	0	0	81	6	0	6	6	16
100	USCγ 2	0	0	80	0	0	0	20	5
28	Cluster 2a, TUSC	0	7	79	0	7	7	0	15
41	Cluster 1l, *Crenothrix* related	0	0	75	13	13	0	0	8

OTUs are defined as habitat-specific if at least 75% of the non-redundant reads were detected in one habitat. Common OTUs were detected in at least five different habitats. The group of upland soils includes hydromorphic soils, arctic-alpine soils, volcanic soils and polluted soils. Cultivated OTUs contain at least one sequence of a cultivated strain, but not necessarily a type strain. Color coding reflects relative detection frequency across habitats.

**Table 9:** **Broadly distributed and habitat-specific OTUs_4_**.

**OTU**	**Name of cluster**	**Marine**	**Aquatic**	**Soil, weak methane supply**	**Rice**	**Wetland**	**Bog**	**Soil, landfill**	**Number of non-redundant sequences**
**COMMON, CULTIVATED**									
1	*Methylocystis echinoides*	2	24	16	47	4	2	4	47
3	*Methylocystis rosea, hirsuta*	0	29	26	19	6	5	15	93
10	*Methylocystis* sp.	0	11	14	68	4	0	4	30
28	*Methylosarcina lacus*	9	27	9	45	0	0	9	12
40	*Methylobacter tundripaludum*	0	20	10	0	30	10	30	10
127	*Methylocystis sp., pmoA2*	0	50	17	8	0	8	17	12
**COMMON, UNCULTIVATED**									
25	*Methylosarcina*	0	0	33	33	8	0	25	12
32	*Methylosoma*	0	17	17	42	17	0	8	13
41	RCL a	0	8	33	8	8	0	42	12
59	*Methylocystis*	0	14	29	0	14	29	14	7
73	*Methylocystis*	0	10	10	30	10	30	10	10
**SPECIFIC, CULTIVATED**									
94	*Methylobacter* sp.	0	100	0	0	0	0	0	8
137	*Methylocystis* sp.	0	86	0	14	0	0	0	7
43	*Methyloparacoccus murrellii*	11	78	0	0	11	0	0	9
376	*“Methylacidiphilum fumarolicum, kamchatkense,“ pmoA2*	0	0	100	0	0	0	0	5
149	*Methylocystis parvus*	0	0	100	0	0	0	0	7
202	*Methylocystis* sp.	0	0	14	0	0	0	86	8
23	*Methylocaldum gracile*	0	8	8	0	0	0	83	13
**SPECIFIC, UNCULTIVATED**									
37	Deep-sea cluster 2r	100	0	0	0	0	0	0	6
19	Deep-sea cluster 3p, OPU3	100	0	0	0	0	0	0	5
156	Deep-sea cluster 3p, OPU3	100	0	0	0	0	0	0	5
42	Deep-sea cluster 5w, OPU1	100	0	0	0	0	0	0	6
64	Aquatic cluster 2b	0	100	0	0	0	0	0	5
61	Aquatic cluster 4a	0	100	0	0	0	0	0	8
7	Lake cluster 1a	0	100	0	0	0	0	0	9
350	*Methylobacter psychrophilus*	0	100	0	0	0	0	0	5
229	*Methyloglobulus* like 13, LP20	0	100	0	0	0	0	0	6
197	*Methylomonas*	0	100	0	0	0	0	0	5
52	*Methyloparacoccus*	0	100	0	0	0	0	0	5
158	*Methyloparacoccus*	0	100	0	0	0	0	0	7
332	*Methylosoma*	0	100	0	0	0	0	0	6
50	FWs 1a	0	80	20	0	0	0	0	5
270	*Methylocystis*	0	80	0	0	0	20	0	5
2	USCα 4, RA14	0	0	100	0	0	0	0	20
58	USCα 4, RA14	0	0	100	0	0	0	0	7
140	USCα 4, RA14	0	0	100	0	0	0	0	5
90	USCγ 1	0	0	100	0	0	0	0	6
33	USCα 16, JR1, Cluster 5	0	0	88	13	0	0	0	8
205	USCγ 1	0	0	80	0	0	0	20	5
242	*Methylococcaceae*	0	0	0	100	0	0	0	5
54	*Methylocystaceae* 11	0	0	0	100	0	0	0	6
343	*Methylocystis*	0	0	0	100	0	0	0	5
14	*Methylomonas*	0	0	0	100	0	0	0	8
150	*Methyloparacoccus*	0	0	0	100	0	0	0	5
65	RPC 2a	0	0	0	100	0	0	0	10
97	RPC1_3 like 10, RPC1	0	0	0	100	0	0	0	7
95	RPC 2a	0	0	7	93	0	0	0	14
26	*Methyloparacoccus*	0	0	9	91	0	0	0	11
102	*Methylocystis*	0	0	14	86	0	0	0	7
9	*Methylosarcina*	0	0	14	82	0	0	5	23
130	*Methyloparacoccus*	0	0	9	82	0	0	9	12
48	*Methylosarcina*	0	11	11	78	0	0	0	9
387	*Methylocystis*	0	0	0	0	0	100	0	5

OTUs are defined as habitat-specific if at least 75% of the non-redundant reads were detected in one habitat. Common OTUs were detected in at least five different habitats. The group of upland soils includes hydromorphic soils, arctic-alpine soils, volcanic soils, and polluted soils. Cultivated OTUs contain at least one sequence of a cultivated strain, but this is not necessarily a type strain. Color coding reflects relative detection frequency across habitats.

The marine habitats, which appeared most distinct in the NMDS plots, are not only characterized by the presence of very unique taxa that are mostly absent from all other ecosystems. Additionally, most taxa with broad distribution in diverse habitats are largely absent in marine ecosystems, in particular at species level resolution (Figure 8). The OTUs that are characteristic for marine habitats belong to the deep-sea clusters 1 to 5 (Table 8). Due to a high phylogenetic diversity within these clusters, most of the individual OTUs have so far only been detected in a few studies, so that many OTUs of these clades were excluded from this kind of analysis. This explains the unexpectedly low number of OTUs that are displayed for the marine samples at species level resolution in the heat map (Figure 8).

The different aquatic habitats have several OTUs at genus and species level in common (Figure 8). This includes the uncultivated clusters FWs, lake cluster 1 and LP20, which have already been described as habitat-specific before (Dumont et al., 2014), and most of the aquatic clusters that were defined in this article. Moreover, the genus *Methyloparacoccus murrellii* as well as specific OTUs_4_ of the genera *Methylobacter, Methylomonas, Methylosoma*, and *Methylocystis* are characteristic for aquatic habitats (Table 9). Some OTUs are even more habitat specific and occur preferentially either in the water column or the sediment. Specific for the water column are the aquatic clusters 2b and 5a and lake cluster 1, while aquatic cluster 4a, the *Methyloglobulus* like cluster LP20 and some further OTUs related to M*ethylobacter psychrophilus, Methyloglobulus morosus, Methyloparacoccus murrellii* and *Methylosoma difficile* are specific for the sediment (Figure 8, Tables S1–S4). In agreement with this preferential occurrence, the cultivated strains of these species were also obtained from aquatic habitats (Table 2).

The terrestrial habitats show a lower number of specific OTUs, in agreement with the weaker resolution in the NMDS plots. Rice associated habitats harbor no characteristic OTUs at genus level resolution, but some specific OTUs related to *Methyloparacoccus* and *Methylocystis* or the uncultivated lineages RPC1 and RPC2 at higher taxonomic resolution (Table 9). Characteristic in landfill cover soils are strains of *Methylocaldum gracile* and of an unclassified *Methylocystis* species, but no specific clusters of uncultivated methanotrophs were detected. As expected, different lineages of USCα and USCγ are specific for upland soils, while the genus *Methylocapsa* and a specific uncultivated *Methylocystis* species are typical inhabitants of bog ecosystems (Tables 8, 9).

### Broadly distributed methanotrophic taxa

Several OTUs_12_ occur in diverse habitats. These include a number of cultivated genera, in particularly those that have been discovered and described quite early and that have been obtained as isolates frequently (Tables 4, 8). Furthermore, some lineages of uncultivated methanotrophs are broadly distributed such as OTUs of the clusters FWs, RCL, RA21, or RPC1_3. At species level resolution, the number of common OTUs is lower (Table 9). This can be explained by habitat specialization with increasing taxonomic resolution, as observed for cultivated and uncultivated members of the genera *Methylocystis, Methylocaldum*, or *Methylobacter* (Tables 8, 9).

## Understanding the influence of environmental factors on methanotrophic community composition and activity

It is obvious that the occurrence and activity of methanotrophic bacteria in different ecosystems is largely influenced by abiotic and biotic environmental conditions. Important factors are methane and oxygen concentrations, nutrient availability, pH, temperature, salinity and water availability (Semrau et al., 2010). Additional factors will influence these bacteria indirectly such as soil moisture content, which affects gas diffusion and thus methane and oxygen supply, or plant cover, which alters the water and nutrient status in soil. Among these factors, methane concentration, nitrogen status and the role of copper have been studied in most detail and were identified as very important for shaping methanotrophic communities and for influencing their activity (Conrad, 2007; Semrau et al., 2010; Ho et al., 2013). Future research needs to address the question how the different factors act alone and in combination on the members of methanotrophic communities in different ecosystems. The present study evaluated only the presence or absence of methanotrophic bacteria in the different ecosystems, but this does not implement information about metabolic activity. In particular type IIa methanotrophs are capable of forming resting stages, which enable prolonged survival under unfavorable conditions (Whittenbury et al., 1970a). To link ecosystem function with community composition, activity in dependence on environmental parameters needs to be analyzed in more detail in future studies.

The present review provides a comprehensive overview about habitat preferences of methanotrophic taxa, considering the complete diversity as represented by *pmoA* as marker and including all major ecosystems in which these bacteria occur. However, habitat preferences do also exist within these ecosystems. The preferential occurrence of USCα in acidic and USCγ in pH neutral upland soils or the plant genotype specific colonization of rice by uncultivated groups of methanotrophs are just two examples (Knief et al., 2003; Lüke et al., 2011). In the latter case, differences can be seen as shifts in the methanotrophic community composition, but not based on pure presence absence data. Likewise, shifts have been observed in aquatic ecosystems, where methanotrophic communities differ in dependence on depth or type of sediment (Pester et al., 2004; Rahalkar and Schink, 2007; Biderre-Petit et al., 2011; Deutzmann et al., 2011). In contrast, almost nothing is known about niche differentiation and habitat preferences among all those OTUs that represent uncultivated genera and species of the marine deep-sea clusters. These methanotrophs appear to coexist in marine habitats, or differentiation occurs at a finer scale. In-depth studies within the different ecosystems are needed to obtain further knowledge about habitat preferences of the individual clusters of methanotrophic bacteria. Such studies need to implement meta-data describing the physicochemical and biological characteristics of the habitat or have to be done under controlled conditions whereby specific parameters are manipulated.

There is a clear need to study the impact of environmental factors at different taxonomic resolution in order to gain comprehensive understanding about mechanisms that lead to niche differentiation among methanotrophs. In initial studies, a simple differentiation between type I and type II methanotrophs was made (e.g., Graham et al., 1993; Amaral et al., 1995; Henckel et al., 2000b), which is certainly appropriate due to some major differences that exist between these groups, e.g., in terms of physiology. Hence, these studies provided valuable insight concerning the differential responses of the studied methanotrophs to high and low methane, oxygen and nitrogen concentrations (Conrad, 2007; Ho et al., 2013). However, the compilation of ecophysiological characteristics from type strains in this study has shown that responses of methanotrophic bacteria to specific environmental factors are often not closely linked to phylogeny, a finding that was recently also reported by Krause et al. (2014), so that other approaches may be necessary to categorize methanotrophs. A concept that has several times been applied considers type I methanotrophs as r-strategists and type II methanotrophs as k-strategists (Steenbergh et al., 2010; Siljanen et al., 2011). A recent proposition is based on a classification of methanotrophic bacteria into more specific ecological response groups based on specific functional traits: methanotrophic genera were classified based on their life strategies as competitors, stress tolerators or ruderals (Bodelier et al., 2012; Ho et al., 2013). The data compiled in this review clearly support the assumption that methanotrophic bacteria have developed different life strategies. Several groups of methanotrophs, among them many uncultivated lineages, appear to be specifically adapted to a certain habitat type and may thus represent good competitors in this specific environment. Some others have been found more widespread in different habitat types and may thus represent stress tolerators and/or ruderals.

## Importance to obtain further isolates of methanotrophic bacteria

The evaluation of the representativeness of cultured model strains has revealed that they cover already a substantial fraction of the frequently detected methanotrophs in environmental samples. Several of them are common colonizers in diverse habitats. This encompasses in particular those taxa that are easily recovered in enrichment studies, while other isolated species and genera have not (yet) been frequently detected in nature. The fact that major clusters of uncultivated methanotrophs are detected in diverse ecosystems clearly shows the need for further isolation efforts to get hands on these organisms. This applies in particular to the frequently detected methanotrophs belonging to the diverse rice paddy clusters, the marine deep-sea clusters, the upland soil clusters or the different aquatic clusters. It is likely that these organisms are well adapted to their respective habitats, so that specific enrichment strategies may have to be applied, which better mimic the natural conditions of these methanotrophs to stimulate their growth. Several attempts were already made to enrich USCα methanotrophs, but until now, these resulted in the retrieval of well-known methanotrophic genera such as *Methylocystis* and *Methylosinus* rather than an enrichment of bacteria harboring USCα gene sequences (Dunfield et al., 1999; Knief and Dunfield, 2005; Kravchenko et al., 2010).

Only the combination of community analyses in natural environments, under controlled conditions in microcosms or mesocosms and of pure cultures or enrichment cultures will allow to understand the physiological and regulatory mechanisms at cellular level that ultimately control activity and affect dispersal of methanotrophs in nature. The fact that many gene functions and regulatory mechanisms in methanotrophic bacteria are until now only little understood, e.g., the role of *pxmA*, limits also the gain of knowledge from cultivation-independent studies when global analysis approaches such as metagenomics, -transcriptomics or -proteomics are applied. This underlines the need for studying pure cultures under laboratory conditions.

The analysis of dispersal patterns at high taxonomic resolution needs a sufficiently large data basis. Conclusions about habitat preferences can only be drawn for frequently detected genera and species, but not so easily for those methanotrophic genera that are currently represented by a single strain or a very small number of sequences. Their less frequent recovery in cultivation-dependent and -independent approaches might point toward higher specialization. In order to draw further conclusions about habitat preferences for these smaller groups, the detection of similar sequences in cultivation-independent studies and/or the isolation of further representatives are necessary. The application of next generation sequencing techniques will facilitate the detection of such rare methanotrophs due to the higher sequencing depth that can be reached. However, currently the integration of NGS data from studies into existing sequence databases is time consuming, as tools for data mining are still largely lacking. At the moment, this limitation can most conveniently be overcome if authors deposit representative *pmoA* sequences in the NCBI nucleotide database or provide them as fasta files. NGS sequencing technology is more and more frequently applied to characterize methanotrophic communities and will lead to an enormous amount of data in the next years. If these data are supplemented with detailed information about the sampling sites and the experimental conditions, it may become a very valuable data resource, enabling more detailed meta-analyses, focusing on specific ecosystems, environmental factors, or taxonomic groups.

### Conflict of interest statement

The author declares that the research was conducted in the absence of any commercial or financial relationships that could be construed as a potential conflict of interest.
